# Electrospun Poly(vinyl alcohol)-Based Conductive Semi-interpenetrating Polymer Network Fibrous Hydrogel: A Toolbox for Optimal Cross-Linking

**DOI:** 10.1021/acsmaterialsau.3c00025

**Published:** 2023-06-08

**Authors:** Anna Zakrzewska, Seyed Shahrooz Zargarian, Chiara Rinoldi, Arkadiusz Gradys, Dariusz Jarząbek, Michele Zanoni, Chiara Gualandi, Massimiliano Lanzi, Filippo Pierini

**Affiliations:** † Department of Biosystems and Soft Matter, Institute of Fundamental Technological Research, Polish Academy of Sciences, Pawińskiego 5B, 02-106 Warsaw, Poland; § Laboratory of Polymers and Biomaterials, Institute of Fundamental Technological Research, Polish Academy of Sciences, Pawińskiego 5B, 02-106 Warsaw, Poland; ⊥ Department of Mechanics of Materials, Institute of Fundamental Technological Research, Polish Academy of Sciences, Pawińskiego 5B, 02-106 Warsaw, Poland; ∥ Department of Chemistry “Giacomo Ciamician″, University of Bologna, Via Selmi 2, 40126 Bologna, Italy; ‡ Department of Industrial Chemistry “Toso Montanari”, University of Bologna, Viale del Risorgimento 4, 40136 Bologna, Italy

**Keywords:** poly(vinyl alcohol), poly[3-(potassium-5-butanoate)thiophene-2,5-diyl], electrospun nanofibers, cross-linking, fibrous hydrogel, semi-IPN

## Abstract

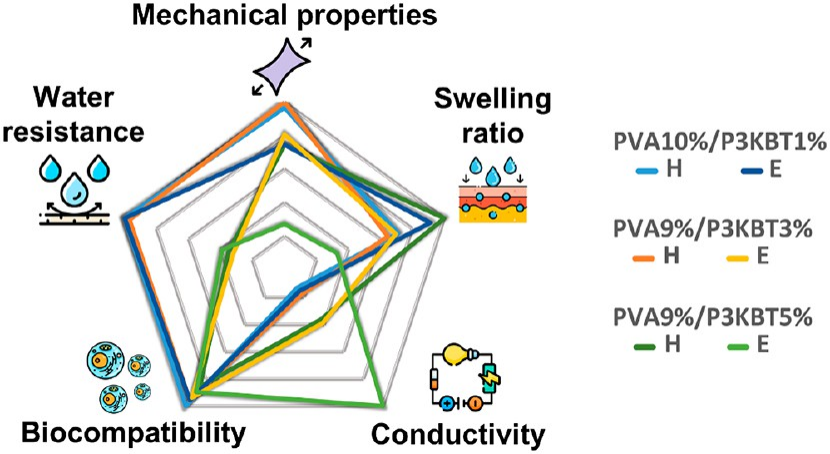

Cross-linking of poly(vinyl alcohol) (PVA) creates a three-dimensional network by bonding adjacent polymer chains. The cross-linked structure, upon immersion in water, turns into a hydrogel, which exhibits unique absorption properties due to the presence of hydrophilic groups within the PVA polymer chains and, simultaneously, ceases to be soluble in water. The properties of PVA can be adjusted by chemical modification or blending with other substances, such as polymers, *e.g*., conductive poly[3-(potassium-5-butanoate)thiophene-2,5-diyl] (P3KBT). In this work, PVA-based conductive semi-interpenetrating polymer networks (semi-IPNs) are successfully fabricated. The systems are obtained as a result of electrospinning of PVA/P3KBT precursor solutions with different polymer concentrations and then cross-linking using “green”, environmentally safe methods. One approach consists of thermal treatment (H), while the second approach combines stabilization with ethanol and heating (E). The comprehensive characterization allows to evaluate the correlation between the cross-linking methods and properties of nanofibrous hydrogels. While both methods are successful, the cross-linking density is higher in the thermally cross-linked samples, resulting in lower conductivity and swelling ratio compared to the E-treated samples. Moreover, the H-cross-linked systems have better mechanical properties—lower stiffness and greater tensile strength. All the tested systems are biocompatible, and interestingly, due to the presence of P3KBT, they show photoresponsivity to solar radiation generated by the simulator. The results indicate that both methods of PVA cross-linking are highly effective and can be applied to a specific system depending on the target, *e.g*., biomedical or electronic applications.

## Introduction

Poly(vinyl alcohol) (PVA) is a water-soluble synthetic polymer^1^ used in various applications due to its unique properties^2^ such as hydrophilicity,^3^ high solubility in polar solvents,^4^ chemical stability,^5,6^ and temperature resistance.^7^ One of its synthesis methods is based on poly(vinyl acetate) hydrolysis in the presence of bases, which leads to the removal of acetate ester groups from the polymer chain and the formation of alcohol groups.^7−9^

The degree of hydrolysis of the PVA is expressed as the percentage of acetate groups that have been cleaved from the polymer. Fully hydrolyzed PVA has a degree of hydrolysis of around 99%, meaning that almost all acetate groups have been removed from the polymer chain. This type of PVA is soluble in hot water.^6^ Partially hydrolyzed PVAs can also be found on the market; in this frame, the most common one has a degree of hydrolysis between 87% and 89%. This partially hydrolyzed PVA is soluble in water at room temperature^6^ and known for its uses in food packaging.^9^ Besides, PVA has found application in many other fields.^10^

It is worth noting that PVA is a highly spinnable polymer,^11^ quite often used for the production of nanofibrous materials.^12^ Recently, electrospinning, which is a technique that allows the fabrication of continuous nanofibers,^13^ has been of great interest to scientists. The basic principle of this technique is to spray a polymer solution under the influence of an electric field. A voltage is applied to the metal needle of a solution-filled syringe, resulting in the ejection and stretching of a liquid jet and, finally, the deposition of nanofibers on the grounded collector surface.^14,15^

Electrospun PVA nanofibers have many applications, especially in industry and medicine. They are used as filter materials,^12,16−18^ in textiles,^19−21^ and as drug carriers.^22−26^ Moreover, PVA nanofibers are biodegradable and biocompatible, have low toxicity,^27^ and can be functionalized with different chemical groups.^28^

In order to improve mechanical properties and chemical resistance, as well as to reduce water solubility, which is crucial from the point of view of many polymer applications, cross-linking is used.^29^ This process involves bonding adjacent polymer chains, resulting in the creation of a three-dimensional network. Once submerged in water, the cross-linked structure transforms into a hydrogel with high water absorbency. This phenomenon can be attributed to the presence of hydrophilic groups within the PVA polymer chains.^30,31^ Many scientists reported various methods of PVA cross-linking, such as exposure to glutaraldehyde vapors^32−35^ or immersion in a solution of glutaraldehyde.^31,36−38^ There are also more and more popular so-called “green” methods of PVA cross-linking, which allow avoiding the use of toxic and harmful substances to the environment. “Green” approaches encompass the use of high temperatures,^35,39−41^ UV radiation treatment,^35,42^ freezing and thawing cycles,^43,44^ stabilization with methanol,^35,41^ immersion in ethanol combined with thermal tratment,^45^ or the addition of citric acid.^46,47^ The cross-linked fibers result in a nanofibrous hydrogel with a three-dimensional structure,^48^ large specific surface area, and high free volume.^46^ This provides its unique absorption properties^44^ for water and other chemicals, as well as particles of various sizes, which opens the way to use of cross-linked nanofibers in the field of filtration.^49^ Nonetheless, whether residing in its dry or hydrogel state, PVA lacks electrical conductivity, which limits its use in electronic applications. To overcome this limitation, PVA can be chemically modified, *e.g*., by introducing functional groups that will improve the electrical properties of the material. Another way may be the addition of a second, specific substance that is intrinsically conductive, such as carbon nanotubes, graphene, or a conducting polymer.

Regioregular poly[3-(potassium-5-butanoate)thiophene-2,5-diyl] (P3KBT) is an organic semiconductive polymer composed of thiophene monomer with butanoate and potassium side chains.^50^ P3KBT was first described in 2005 by a group of scientists from the University of Beijing, who developed an innovative method of its synthesis using a palladium catalyst. Regioregularity means that, during synthesis, monomers connect in such a way that long polymer chains have a uniform spatial orientation.^51^ The electronic properties of regioregular polymers are better than those with disordered monomeric units.^52^ Due to their ability to conduct electric current and light absorption in the visible range, these polymers are widely used in organic electronics and photovoltaics.^53^ So far, various biocompatible systems reacting to NIR radiation have been studied developing *e.g*., smart nanostructured pillow for drug delivery application^54^ and glucose-sensing system^55^ based on PNIPAAm-derivative plasmonic hydrogel. P3KBT seems to be another promising polymer worthy of detailed analysis in the field of photoresponsive materials. Moreover, P3KBT is electrospinnable,^56^ relatively thermally stable, and can react with various chemical compounds; unlike standard polythiophene, it is soluble in water.^57^

Combining two or more polymer networks into one interconnected structure with no presence of covalent bonds is called interpenetrating polymer network (IPN).^58^ In IPN, the individual networks cannot be easily separated, and both polymers are cross-linked.^44^ Another type of polymer network is semi-IPN, **i.e.**, a combination of two polymers, but only one of them is cross-linked, while the other remains in its linear molecular form.^58^ The non-cross-linked polymer is thus physically trapped into the cross-linked network. The unique advantage of these materials is that both semi-IPN-forming polymers can be selected independently; therefore, tailoring the material properties to the intended purpose is relatively simple, and the possibilities are endless. Importantly, P3KBT is a polymer that cannot be cross-linked by simple methods but conducts electric current, so it can be used to adjust the properties of another polymer, such as cross-linkable PVA.

In this work, PVA/P3KBT nanoplatforms were developed and fabricated by electrospinning and then stabilized using two “green” cross-linking methods—thermal treatment and immersion in ethanol combined with heating. This resulted in fibrous hydrogels composed of cross-linked PVA polymer networks where P3KBT is trapped inside. Therefore, the obtained system represents a semi-IPN. The nanoplatforms were tested for morphology, stability in water, and chemo-physical features. Photoresponsivity tests were also carried out, and the electrical properties, interactions with water, and mechanical resistance of the samples were investigated. Finally, cell studies were performed to determine the biocompatibility of the systems. All these efforts were made to finally ensure a toolbox for optimal cross-linking of PVA-based nanofibrous systems, as well as to demonstrate their usefulness and potential for application in many different and contrasting fields of science like biomedical engineering—filters, dressings, implants—and electronic devices or photovoltaics.

## Experimental Section

### Materials

PVA with an average molecular weight (Mw) in the range of 85 000–124 000 Da and a degree of hydrolysis (DH) of 99+%, molecular iodine (I_2_), L929 murine fibroblasts, bovine serum albumin (BSA), hexamethyldisilazane (HMDS), phosphate buffer saline (PBS), glutaraldehyde (GTA), Triton X, and DAPI were obtained from Sigma-Aldrich (Poland). P3KBT and ethanol were purchased from Rieke Metals (USA) and Chempur (Poland), respectively. Dulbecco’s modified Eagle’s medium (DMEM), fetal bovine serum (FBS), penicillin-streptomycin (PS), and EDTA-trypsin were bought from Gibco Invitrogen (USA). Alexa Fluor 488 Phalloidin and PrestoBlue reagent were purchased from Thermo-Fisher Scientific (USA).

### Methods

#### Fabrication of PVA and PVA/P3KBT Systems by Electrospinning

PVA solutions containing a water-soluble P3KBT, *i.e.*, semi-IPN precursors, were prepared by mixing aqueous PVA solutions with an aqueous P3KBT solution in various proportions to obtain membranes with different PVA/P3KBT compositions. In a typical preparation procedure, PVA was first mixed in Milli-Q deionized water and stirred at 80 °C for 4 h until complete dissolution. The concentration of PVA was adjusted for obtaining 9% or 10% (w/v) in the final solution (after mixing it with the proper volume of P3KBT solution). PVA concentrations were selected experimentally as shown in Figure S1. P3KBT was also dissolved in deionized water (2% w/v) and stirred for 24 h before being added to the PVA solutions. Solutions contained pure PVA 9% and 10% (w/v), without the addition of P3KBT, were used to fabricate nanofibers, and to carry out some analysis for comparative purposes. The prepared precursor solutions contained the following concentrations of polymers:

- PVA 10%/P3KBT 0.00% (w/v); P3KBT/PVA = 0%,
- PVA 10%/P3KBT 0.10% (w/v); P3KBT/PVA = 1%,
- PVA 10%/P3KBT 0.20% (w/v); P3KBT/PVA = 2%,
- PVA 10%/P3KBT 0.30% (w/v); P3KBT/PVA = 3%,
- PVA 9%/P3KBT 0.00% (w/v); P3KBT/PVA = 0%,
- PVA 9%/P3KBT 0.27% (w/v); P3KBT/PVA = 3%,
- PVA 9%/P3KBT 0.36% (w/v); P3KBT/PVA = 4%,
- PVA 9%/P3KBT 0.45% (w/v); P3KBT/PVA = 5%.

Immediately before electrospinning, ethanol was adding to the polymer mixture in a volume ratio of 1:9. Each electrospinning solution was placed in a 1 mL syringe with a 26G needle (outer diameter = 0.45 mm). The solutions were spun onto a flat or drum collector under conditions optimized accordingly to the PVA/P3KBT ratio. The distance between the needle and the collector varied in the range of 12–15 cm, the voltage was in the range of 13–15 kV, while the flow rate of the solutions was set at 500 μL h^–1^ (Table S1).

Thereafter, electrospun materials are coded based on the concentration of PVA and the P3KBT/PVA ratio in subsequent precursor solutions as follows:

- PVA10%/P3KBT0%,
- PVA10%/P3KBT1%,
- PVA10%/P3KBT2%,
- PVA10%/P3KBT3%,
- PVA9%/P3KBT0%,
- PVA9%/P3KBT3%
- PVA9%/P3KBT4%,
- PVA9%/P3KBT5%.

Precursor solution and sample details are provided in Table S2.

#### Nanofiber Cross-Linking

Two different methods were selected and used to cross-link the electrospun nanofibers (Figure S2). The first method involved heating the samples in an oven at 160 °C for 2 h (H), while the second method involved immersing the samples in ethanol for 24 h, followed by air-drying and thermal treatment at 160 °C for 20 min (E).

### Morphological Characterization

Each sample, both non-cross-linked (nc) and cross-linked (H, E), was imaged using a scanning electron microscope (JSM-6010PLUS/LV, In TouchScope microscope). Before imaging, nanofibers were sputtered with gold (2 × 2 min) in a DII-29030SCTR JEOL Smart Coater. All images were taken using the same electron beam energy (7 kV) and the same magnification (×3000).

To precisely determine the diameter of the nanofibers precisely before and after the cross-linking process, FE-SEM imaging was used by operating the ZEISS Crossbeam 350 FIB-SEM microscope. The diameter of the fibers was measured 15 times in different places of the samples. All the images were taken using the SE2 detector, at a magnification of ×10 000 and with an electron beam energy of 5 kV.

### Chemical and Physical Characteristics

Absorption spectra of nanofibrous materials were recorded using a spectrometer for analyzing samples in a solid state (PerkinElmer UV–vis-NIR Lambda 19 spectrometer equipped with a Labsphere RSA-PE-19 Integrating Sphere). For the measurements, mats electrospun onto coverslips with dimensions of 2.4 cm × 2.4 cm were used. The spectra were recorded in the wavelength range of 300–800 nm.

FT-IR analysis was carried out to get information about the chemical structure of fabricated nanofibers in terms of chemical bonds and functional groups. In this study, Vertex70, Bruker spectrometer, and OPUS 8.1 software were used. The samples were scanned from 4000 to 400 cm^–1^ with a resolution of 2 cm^–1^.

Wide angle X-ray diffraction measurements (XRD) were carried out in open air at RT in Bragg-Bentano geometry with a PANalytical X’Pert PRO diffractometer equipped with an XCelerator detector. A Cu anode was used as the X-ray source (λ_1_ = 0.15406 nm, λ_2_ = 0.15443 nm). XRD diffractograms were collected in the 2θ range of 10–50°.

Differential scanning calorimetry (DSC) measurements were performed using power-compensation Pyris 1 DSC calorimeter (PerkinElmer, USA), calibrated with indium and M-24 liquid crystal standards. Samples with masses 2–4 mg were subjected to heating scans at 10 K min^–1^ from −50 to 250 °C. Due to the substantial deviation of the heat flow signal at the end of the high-temperature endothermic peaks, the scans were additionally subtracted using baselines with polynomials approximated with Origin software.

The electrical conductivity of the systems containing P3KBT was tested. Tests were performed using an AlphaLab High Resistance/Low conductance meter (HRLC, AlphaLab Inc.). The nanofibrous samples electrospun onto square coverslips (2.4 cm × 2.4 cm) were doped with I_2_ vapors for 6 h before performing the measurements. Each sample was measured five times, and the results are presented as average values.

Non-cross-linked and cross-linked samples were subjected to tensile strength tests. For this purpose, strips of electrospun nanofibers with dimensions of 4.0 cm × 1.0 cm were prepared. After holding the samples between the grips of the tensile testing instrument, the gauge length was 20 mm. In each test, data were collected at 50 points s^–1^, proper measurement started when the trigger load was 0.10 N, and the measurements were carried out with a speed of 1 mm s^–1^ up to the sample breaking by operating a CTX Texture Analyzer (Brookfield Ametek). Using Texture Pro V1.0 Build 19 software, the force–displacement curves were recorded in triplicates for each condition. The obtained results were converted into stress–strain curves assuming that stress (σ) is the ratio of force to the initial area of a given sample, while a strain (ε) can be defined as the change in sample length compared to its initial length. On this basis, the Young’s modulus (*E*) of each sample was calculated, namely, the ratio between stress (force causing deformation) and strain in the linear elastic range, *i.e.*, its ability to deform under the influence of external forces.

### Nanoplatform Behavior in Water

#### Water Solubility Tests

Two circular samples of similar thickness with a diameter of 1.0 cm were cut out from each mat and placed in separate vials containing 1 mL of deionized water. The vials were sealed, and half of them were left at room temperature for 3 h while the second group of vials was placed in a 39 °C water bath for 3 h. After this time, the water was removed and samples were dried in the air, followed by SEM imaging to visualize any changes in the morphology of the nanofibers. Similar tests were also carried out at room temperature for non-cross-linked samples for comparison.

#### Polythiophene Derivative Release Study

Supernatants from vials in which solubility tests took place were subjected to spectrophotometric analysis (Multiscan GO absorption spectrometer, Thermo Scientific). Measurements were made in the wavelength range of 300–800 nm. Using the same apparatus, a calibration curve for an aqueous solution of polythiophene derivative was determined and used to calculate the amount of P3KTB released in water by the nanofibrous samples.

#### Swelling Ratio

Cross-linked samples, both thermally and by immersion in ethanol combined with heating, were subjected to swelling tests in water. For this purpose, each sample was weighed and then placed in a beaker with deionized water at room temperature for 1 s, 1 min, 3 min, 5 min, 10 min, and 30 min. After removing the samples from the beakers, they were drained of excess water, and their weight was measured again to determine the degree of swelling caused by water absorption.

### Photothermal Responsivity under Solar Irradiation

Due to the presence of polythiophene derivative, the PVA/P3KBT systems absorb radiation with a wavelength of around 500 nm, which is exactly the extreme of solar radiation. To check the photothermal response of nanofibers, radiation generated by the solar simulator (model no. 10500, Abet Technologies, Inc.) and a thermal camera (FLIR A655sc, EC TEST Systems) were used. Nanofibers prepared by electrospinning the same volume of each solution on a drum collector, from which samples of the same size were cut out, were used for these tests. The samples were successively placed under a radiation source operating at 1 kW m^–2^ to simulate the power of the solar radiation. PVA/P3KBT systems were irradiated until the maximum temperature was reached and stabilized, but each analysis lasted no longer than 5 min. Temperature changes were observed using a thermal imaging camera and FLIR Research Studio software. The samples after radiation exposure were analyzed by SEM.

### *In Vitro* Biological Studies

#### Culture of L929 Fibroblast Cells

L929 murine fibroblast cells were cultured in DMEM supplemented with 10% FBS and 1% PS and placed in an incubator at 37 °C and 5% CO_2_. The culture medium was refreshed every 2 days. Cell passaging was performed when the confluence of cells reached ∼80%. For seeding, cells were detached by adding 0.05% EDTA-trypsin for 3 min and incubating the cells at 37 °C and 5% CO_2_. Subsequently, cells were collected in a Falcon tube and centrifuged at 1200 rpm for 5 min. After centrifuging, a pellet of cells was visible at the bottom of the tube. Cells were then resuspended in 1 mL culture medium and counted. Finally, the cell suspension was further diluted in culture media to achieve a convenient cell density for seeding the samples.

#### Sample Sterilization and Seeding

Different concentrations of PVA/P3KBT fibrous samples were electrospun onto coverslips (diameter = 1.5 cm) and cross-linked using the previously discussed methods. Samples were then sterilized by exposure to UV light (30 min cycle for each side). Samples were placed in 24-well plates, and L929 fibroblast cells were seeded on top of the fibrous samples at a density of 10^4^ cells cm^–2^. The control condition was also tested by seeding tissue culture plates (TCP) at the same cell density. All the samples were cultured for up to 7 days.

#### Cell Viability

The viability of cells was measured by PrestoBlue assay. PVA/P3KBT fibrous samples and TCP seeded with L929 fibroblasts were treated with a solution of 10% (v/v) PrestoBlue reagent in culture medium and incubated for 1 h at 37 °C and 5% CO_2_. Five replicates of each sample were analyzed at three selected time points: 1, 3, and 7 days after cell seeding. After 1 h of incubation, 100 μL aliquots of the Prestoblue solution were transferred to a 96-well plate and analyzed at excitation 530 nm and emission at 620 nm by using a fluorometer plate reader (Fluoroskan Ascent TM Microplate Fluorometer, Thermo Scientific).

#### Morphological Evaluation

The morphology of L929 fibroblasts in contact with E- and H-cross-linked PVA9%/P3KBT3% fibrous matrix was evaluated by means of confocal and scanning electron microscopies. Actin staining was performed on three replicates per each condition at 3 and 7 days after cell seeding. Cell cytoskeleton and nuclei were stained by fixing the samples in 4% paraformaldehyde for 15 min at room temperature. Samples were then washed three times in PBS and treated with a solution of 0.3% (v/v) Triton X-100 for 15 min. After washing, a solution of 1% (w/v) BSA in PBS was added to the samples for 30 min. The constructs were incubated in Alexa Fluor 488 Phalloidin solution (1:40) and placed in the dark for 40 min. Lastly, the staining of nuclei was performed by adding 1:500 DAPI solution for 10 min. Samples were finally washed three times in PBS and imaged with a confocal microscope (Leica).

Investigation of the cell morphology via SEM was assessed on triplicates after 7 days of cell culture. Samples were fixed in 3% ice-cold GTA for 3 h. After three washing steps in DI water, samples were dehydrated by soaking for 15 min in solutions with increasing ethanol concentrations: 50%, 70%, 90%, and 100%. Then, HMDS was added to the constructs and samples were dried overnight under a fume hood. Samples were sputter coated with a thin layer of gold and imaged by using a SEM.

### Statistical Analysis

Data are reported in terms of mean ± standard deviation. One-way ANOVA test was assessed and statistically significant differences are reported when p-value ≤0.05: *p ≤ 0.05, **p ≤ 0.01, ***p ≤ 0.001, ****p ≤ 0.0001.

## Results and Discussion

**Figure 1:**
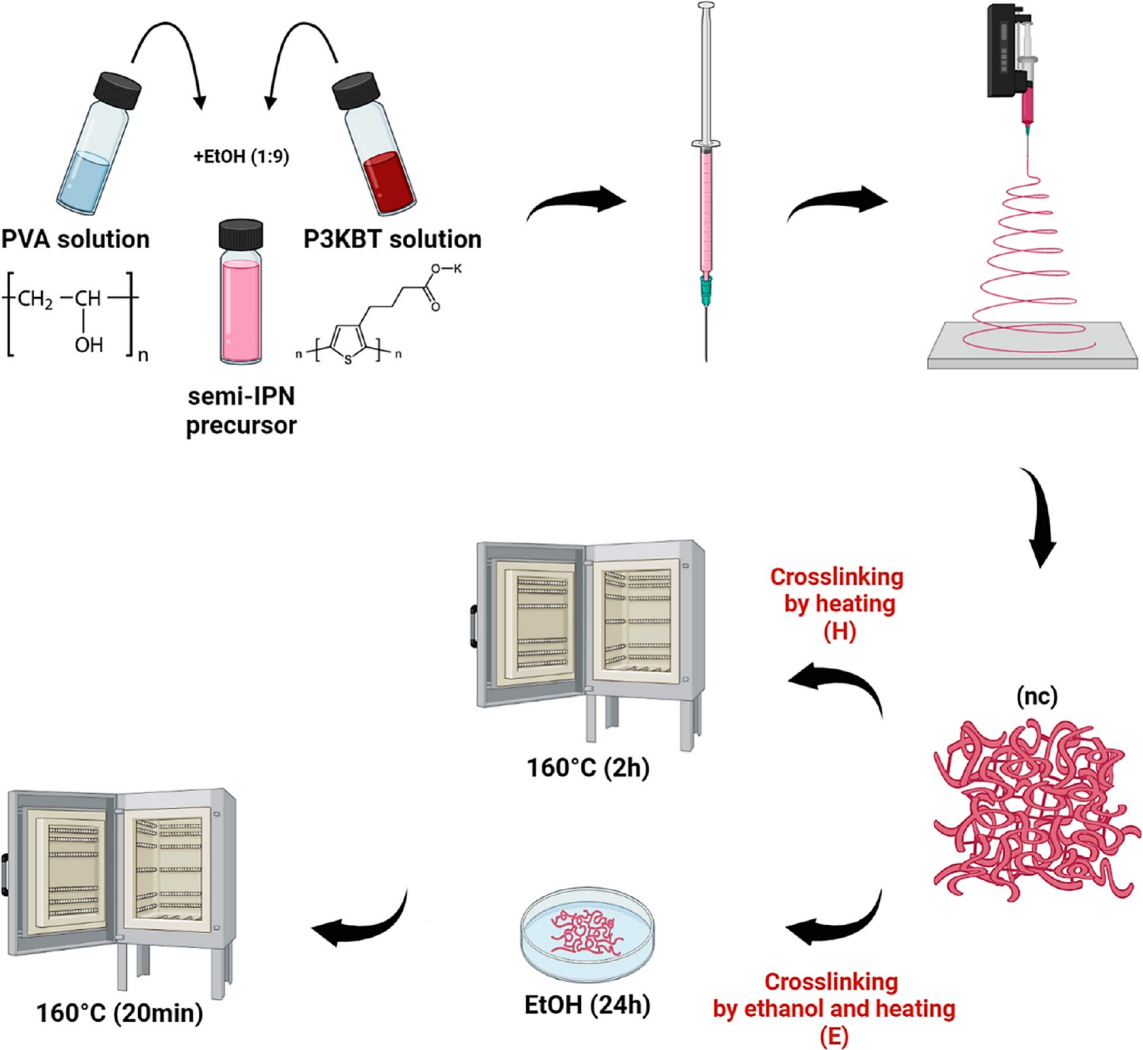
Schematic presentation of the semi-IPN precursor solution preparation and PVA/P3KBT mat (nc) manufacturing process by electrospinning and two “green” cross-linking approaches (H, E). The H method is based on heating only, while the E method includes stabilization with ethanol followed by heat treatment.

The process of PVA/P3KBT nanofibers fabrication by electrospinning and cross-linking using two different methods, heating (H) and immersion in ethanol combined with thermal treatment (E), are schematically shown in Figure 1. The obtained nanoplatforms represent a semi-IPN nanofibrous hydrogels in which PVA is a network-forming polymer, and being trapped inside P3KBT makes the systems electrically conductive and photoresponsive. In this form, PVA/P3KBT nanofibers can be suitable for a wide range of applications, however, selecting effective cross-linking methods and determining their impact on the properties of the semi-IPN is crucial.

### Morphological Characterization

**Figure 2:**
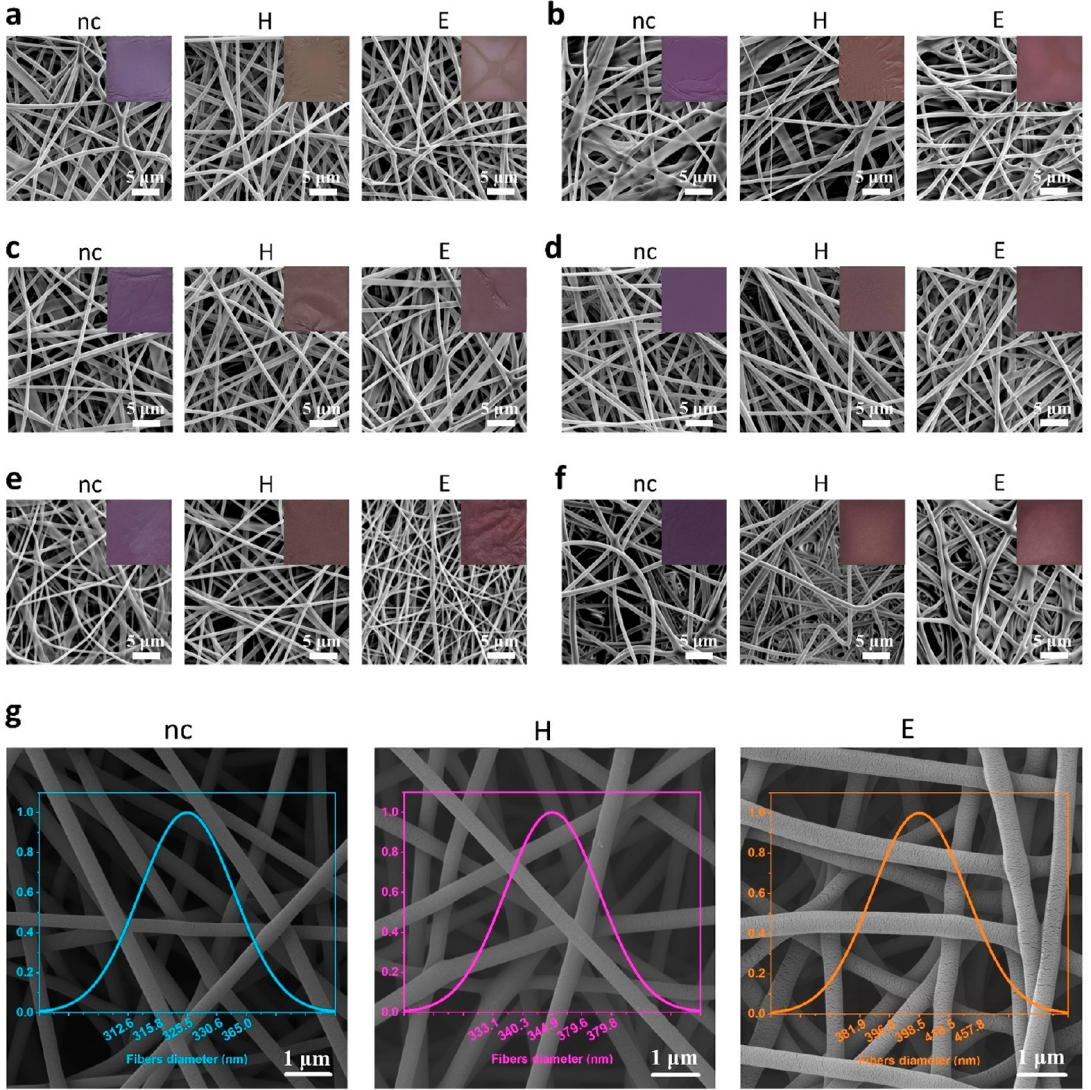
SEM/FE-SEM images and photos of the non-cross-linked (nc) and cross-linked by heating (H) or ethanol immersion combined with heating (E) PVA/P3KBT nanofibers. Dimensions of the photographed samples on the insets (a–f) are 2.4 cm × 2.4 cm. (a) SEM and macroscopic photos of PVA10%/P3KBT1% samples. (b) SEM and macroscopic photos of PVA10%/P3KBT2% samples. (c) SEM and macroscopic photos of PVA10%/P3KBT3% samples. (d) SEM and macroscopic photos of PVA9%/P3KBT3% samples. (e) SEM and macroscopic photos of PVA9%/P3KBT4% samples. (f) SEM and macroscopic photos of PVA9%/P3KBT5% samples. (g) FE-SEM of PVA9%/P3KBT3% mats and distribution of nanofibers diameter among the specific samples. The average diameter of nanofibers was 330 ± 21, 356 ± 22, and 411 ± 29 nm for nc, H, and E sample, respectively. This can be attributed to the formation of specific connections in the cross-linked networks, especially in the case of an E-treated system.

SEM analysis of the nanofibers was performed to study their morphology and changes that occur as a result of cross-linking. Insets in SEM images (Figure 2a–f), showing photos of nanofibrous materials, may suggest that, since high temperature and ethanol cause color changes in the samples, the treatments also affect the morphology of nanofibers. Due to the presence of P3KBT, the obtained mats are purple, and the intensity of this color depends on the P3KBT concentration in a given material. After the stabilization process, all the H-cross-linked samples present color from light to dark brown, while E-treated samples become burgundy. These color differences between systems after cross-linking are probably caused by different high-temperature heating times. SEM images also prove that electrospinning of all solutions resulted in cylindrical, continuous nanofibers with random orientation. The surface of all nanofibers was smooth, without visible beads, although some differences in their diameters within a single sample can sometimes be noticed. The exception to the latter is a PVA9%/P3KBT3% fibrous sample (Figure 2d), which presents the highest degree of homogeneity, as confirmed by the photos in the Figure 2 insets. Both cross-linking processes do not affect the cylindricality of the nanofibers, which is preserved, as well as their smoothness. However, the presented SEM images may suggest that cross-linking affects the fiber diameters. Thus, this supposition has been investigated and discussed.

The FE-SEM analysis was carried out to accurately assess PVA/P3KBT electrospun material with potentially the best morphology homogeneity, *i.e.*, PVA9%/P3KBT3% nanofibers. It was possible to confirm the homogeneous structure of the nanofibers with the desired shape and random orientation, which was indicated by the previously performed SEM analysis. In addition, the FE-SEM technique at a sufficiently high magnification made it possible to determine the diameter of the nanofibers with satisfactory accuracy (Figure 2g). Within a given sample, the diameter of the fibers is comparable, as no significant deviations are observed. In the case of non-cross-linked systems, the average diameter was 330 ± 21 nm, while after cross-linking processes, both the thermal method and the method using ethanol, the diameter of the fibers increased. Its average value in the case of the PVA9%/P3KBT3%H system has been determined as 356 ± 22 nm, and in the case of PVA9%/P3KBT3%E even higher as 411 ± 29 nm.

It is worth recalling that thermal cross-linking is a process in which polymer nanofibers are subjected to heating to bond them together and form a continuous network of polymer chains. During this process, the polymer molecular structure is reorganized, which can affect the diameter of its nanofibers.

In the discussed case, we are probably observing fibers which present specific connections created within their internal structure. These connections between the polymer chain increase nanofibers diameter due to the formation of a more complex structure. The phenomenon of increasing nanofiber diameter is more evident in the case of E method. The chemical step of PVA cross-linking involves ethanol which primarily cross-links the hydroxyl groups of the polymer. In effect, the aqueous hydrogen present in the chain is replaced with intermolecular hydrogen bonds. This causes significant micro- and macrostructural changes, resulting in nanofiber enlargement.^35^

### Chemical and Physical Characteristics

**Figure 3:**
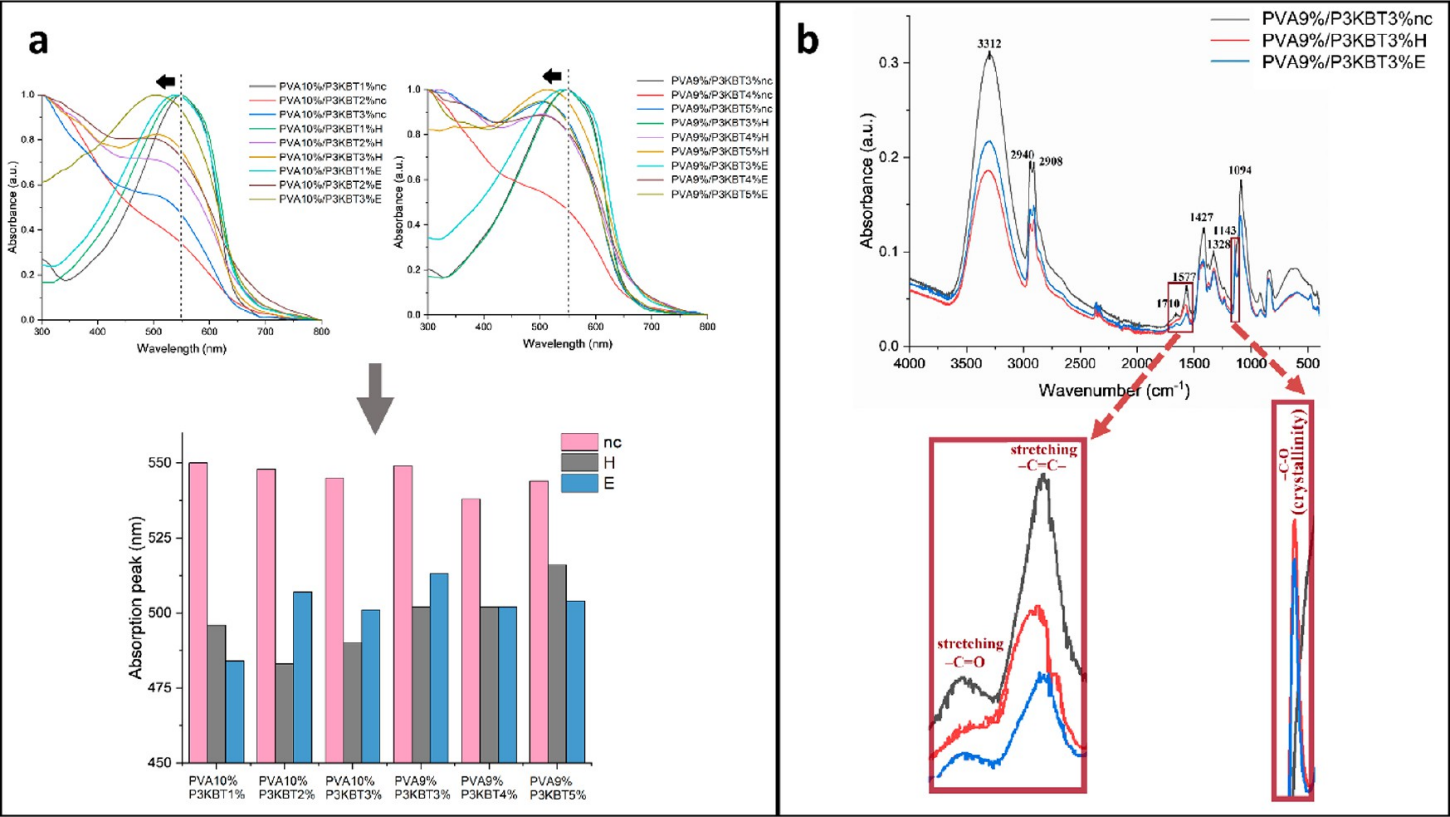
Chemical and physical characteristics of PVA/P3KBT nanofibers. (a) Absorption spectra showing peak shifts and bars representing peak maxima of the analyzed solid samples. (b) FT-IR spectrum of a representative PVA9%/P3KBT3% system before and after cross-linking by H and E methods. The curves in figure (a) were normalized to 1.0. The shift of the absorption spectra toward shorter wavelengths, in the case of cross-linked samples, suggests that interaction between PVA and P3KBT is destroyed as a result of the stabilization process. Changes in the intensity of the peaks in the FT-IR spectrum confirm cross-linking and indicate a higher crystallinity of the H-treated sample compared to the E-cross-linked sample.

The spectra of all nanofibrous materials, differing in PVA and/or P3KBT concentrations, recorded in the wavelength range of 300–800 nm, are shown in Figure 3a. The absorption spectra of solid samples are red-shifted in relation to the spectrum recorded for the aqueous P3KBT solution. This is caused by the amphiphilic nature of PVA and its hydrogen bonds network, as well as the interactions between the two polymers, reducing the aggregation of P3KBT molecules and regulating their conformation. However, among the non-cross-linked samples, a blueshift of the absorption maximum peak toward shorter wavelengths is observed with increasing concentration of the polythiophene derivative. It is known that the number of coplanar rings of polythiophene molecules determines the coupling length. Thus, the increase in coupling length leads to a decrease of the distance between adjacent energy levels and to an increase of the wavelength at which the absorption peak occurs.^59^

It can therefore be assumed that environmental changes, in this case, cause the P3KBT to deviate from coplanarity, which leads to its spine twisting. The reduced conjugation length results in an increase in the distance between the energy levels and, thus, the shortening of the wavelength of the maximum absorption peak. As for the cross-linked samples, there was a shift of the absorption spectra toward shorter wavelengths, even in relation to the aqueous solution. It seems that the high temperature used during cross-linking destroys the interaction between poly(vinyl alcohol) and polythiophene derivative so that the dispersed state and the P3KBT conformation in the dissolved state are not preserved in the solid phase.

The FT-IR spectra recorded for representative samples of PVA9%/P3KBT3% nc, H, and E are shown in Figure 3b. The peaks present in the graph carry crucial pieces of information on the chemical structure of fabricated materials. The broad peak observed from 2988 to 3650 cm^–1^ may be attributed to −O–H stretching due to the strong interaction of the hydrogen bond of the intramolecular and intermolecular characteristics in PVA nanofibers. The peak at 1094 cm^–1^ in the PVA nanofibers is due to −C–O stretching; the cross-linking process was confirmed by the presence of the absorption band indicating the formation of −C–O–C– bonds within the polymer network.^60^ Additionally, the peak at 1143 cm^–1^ allows the crystallinity of the PVA nanofibers to be determined based on the relative intensity of the vibration band. The peak intensity of the H-treated sample, and therefore its crystallinity, is the highest. In general, it can be concluded that the cross-linked materials have a higher degree of crystallinity than the non-cross-linked system, which is explained by the formation of intramolecular hydrogen bonds between two adjacent −O–H groups on the same side of the carbon chain plane.^60^ Furthermore, carbonyl peak stretching vibration at 1710 cm^–1^ and acetal linkage (−C–O–C−) stretching vibrations were identified in the cross-linked PVA/P3KBT nanofibers due to the remaining non-hydrolyzed vinyl acetate group of the PVA. The characteristic absorption bands at 2908 and 2940 cm^–1^ fitted to the asymmetric and symmetric stretching vibration of −CH_2_ groups. The peak at 1427 cm^–1^ appeared due to −CH_2_ wagging, and that at 1328 cm^–1^ is due to −C–H and −O–H bending. Additionally, at 1577 cm^–1^ peak related to −C=C– stretching can be seen. With cross-linking processes, a reduction in absorbance peak intensities at 3312 cm^–1^ is observed, indicating the reduced concentrations of the hydroxyl groups left after the cross-linking reaction. A decrease in the hydroxyl groups results in a loss in the polar nature of the compound, and as a result, the polymer solubility in water decreases. The peak at 1660 cm^–1^ in materials containing polythiophene or its derivative is assigned to the presence of an aromatic ring in the molecule and corresponds to stretching vibrations. A slight increase in the peaks at 1710 cm^–1^ (compare to pure PVA, Figure S3) indicates the formation of ester bonds between the PVA and the P3KBT.

**Figure 4:**
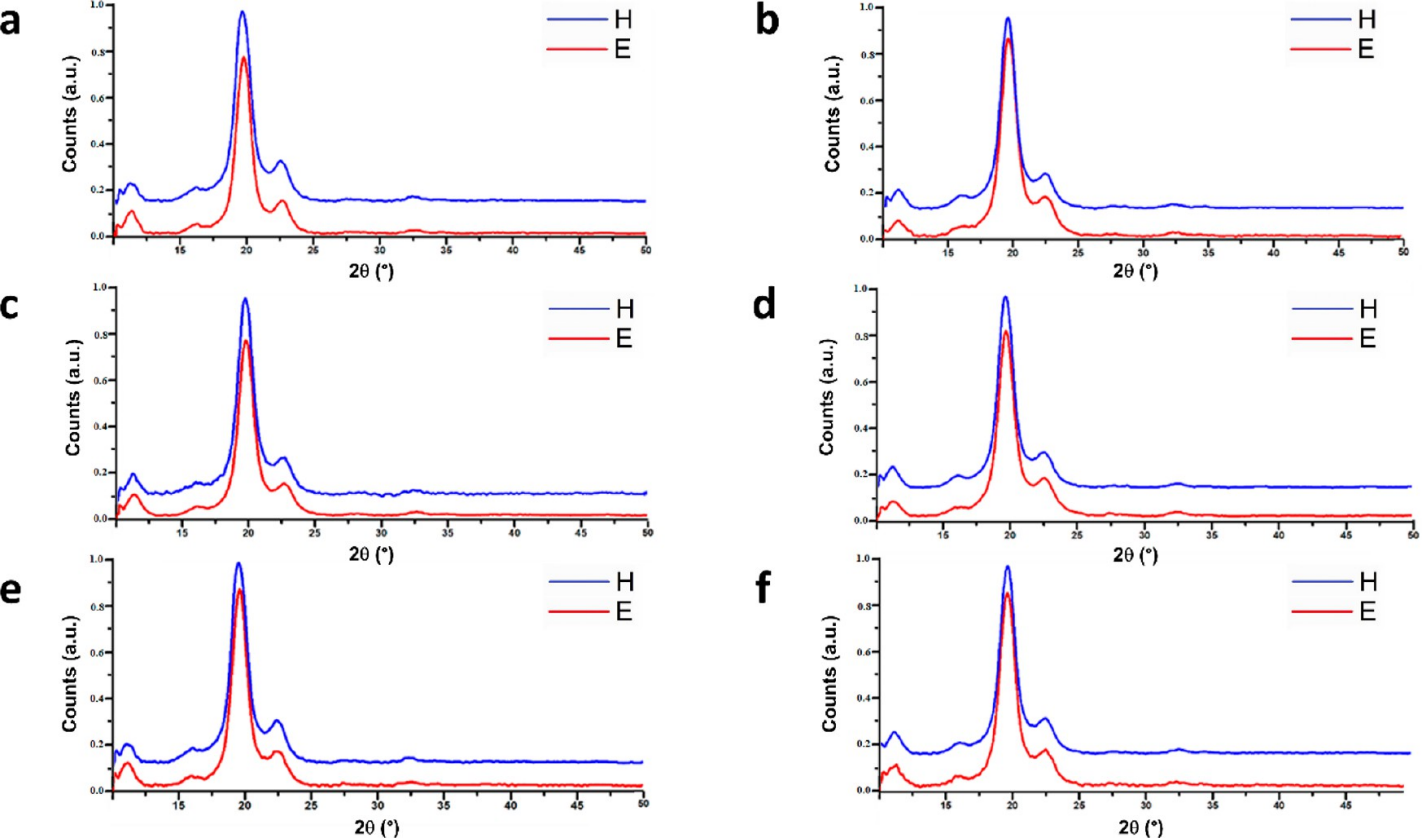
XRD diffractograms of (a) PVA10%/P3KBT1%, (b) PVA10%/P3KBT2%, (c) PVA10%/P3KBT3%, (d) PVA9%/P3KBT3%, (e) PVA9%/P3KBT4%, and (f) PVA9%/P3KBT5%. Blue curves represent patterns of samples cross-linked by the H method, and red curves are related to samples cross-linked by the E method. Estimating the degree of crystal phase indicates 70–80% crystallinity for all samples.

Figure 4 reports the XRD profiles of electrospun samples cross-linked by applying the two different approaches. All samples display the typical reflections of PVA with a sharp and intense reflection at 2θ = 19°, together with less intense peaks located at 2θ = 9°, 11°, 16°, 23° and 32°, 7°, in line with previous results.^61,62^ The XRD pattern is very similar for all samples, and no relevant differences emerge when comparing samples cross-linked by methods E and H. Peak deconvolution allows, in principle, to determine the degree of crystallinity as the ratio of the crystalline peak areas to the total area under the scattering curve. However, the reliable identification of the amorphous halo below the crystalline peaks was not possible and any quantitative measure of the amount of crystal phase would be subject to high uncertainty. A rough estimation of the degree of crystal phase was carried out, resulting in 70–80% crystallinity for all samples.

**Figure 5:**
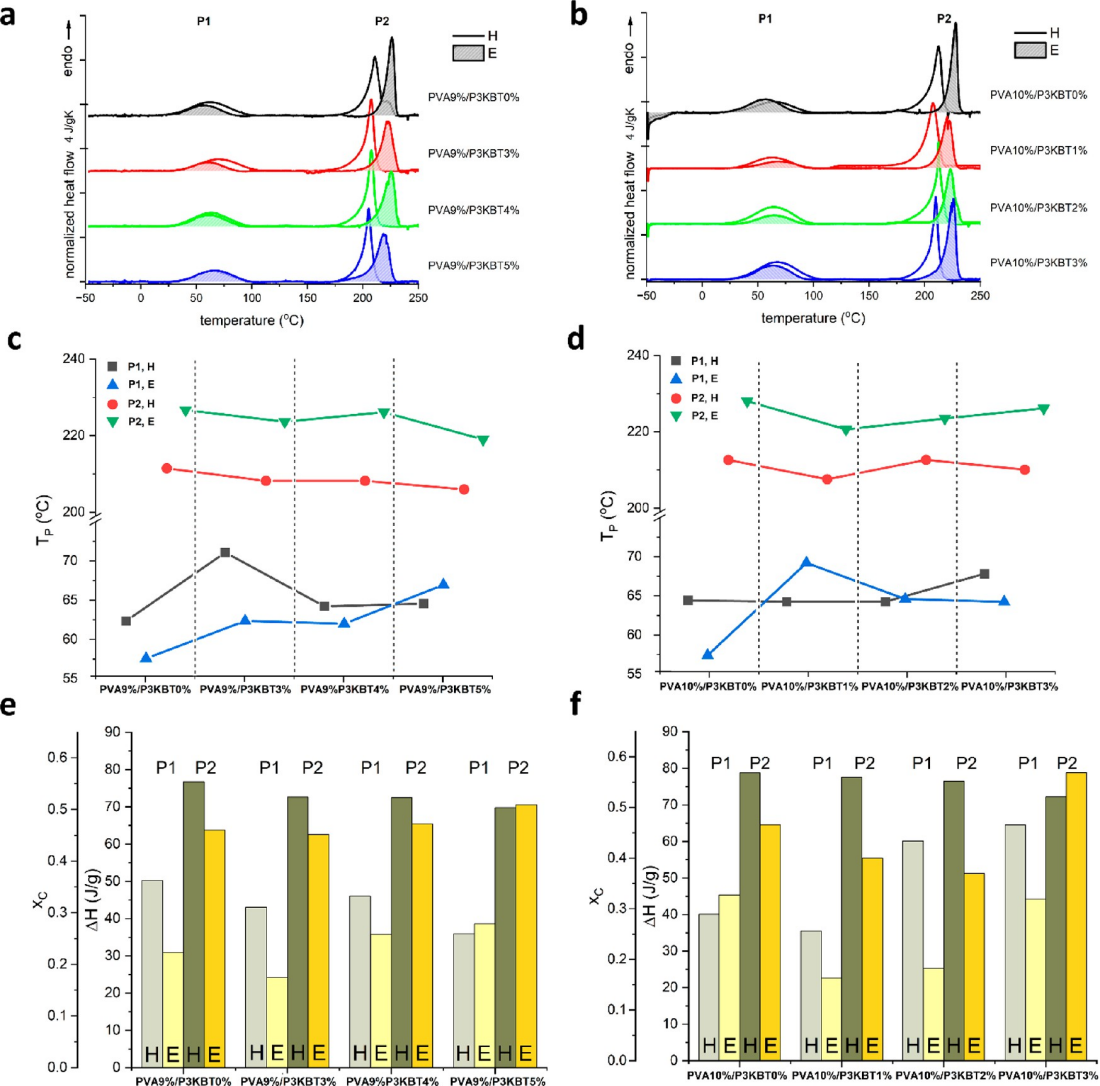
DSC results. (a) Heating scans registered for PVA9%- and (b) PVA10%-based samples. (c) Peak temperature (*T*_p_) for PVA9%- and (d) PVA10%-based samples. (e) Peak area (Δ*H*) and crystallinity (*x*_c_, bars) for PVA9%- and (f) PVA10%-based samples. In (a,b), the curves were shifted for clarity, and they were subtracted with polynomials due to strong heat flow deviation after the high-temperature peak P2. The curves in (a,b) with shaded peaks refer to the E-cross-linked samples. In (c-f), P1 and P2 refer to the low-temperature and the high-temperature peaks, respectively. Crystallinity was calculated using the heat of fusion for 100% crystalline phase, Δ*H*° = 138.6 J g^–1^.^63^

The DSC heating scans registered for all the samples are presented in Figure 5a,b. All scans show two endothermic peaks, a small, broad one located in the range of 60–70 °C, referred to as P1, and an intense, narrow one in the range 200–230 °C, referred to as P2. The peak P1 may be attributed to water evaporation, while the P2 peak is attributed to the crystal melting, with the fusion enthalpy assumed as 138.6 J g^–1^ for the 100% crystalline phase.^63^ It is clearly seen that the melting peaks for the E-cross-linked samples occur at much higher temperatures, near the equilibrium melting temperature of 228 °C, which is plotted in Figure 5c,d. The depression of the melting point in the case of the H-treated samples may be due to higher cross-linking density.^64^ A general observation from Figure 5e,f is higher crystallinities for the H-treated samples with the highest values for PVA10%-based systems. In the Δ*H* data, it may be seen a similar trend for H-cross-linked samples, containing both 9% and 10% of PVA: a decrease in Δ*H* (P2) with an increase in P3KBT concentration. On the other hand, an increasing trend is found for the PVA9%E samples. Interestingly, PVA10%E samples with an increase in P3KBT concentration initially show a decrease and lower Δ*H* for P2 than PVA9%E samples with the exception of highest P3KBT content (PVA10%/P3KBT3%E) which reveal the highest crystallinity, *ca*. 0.57. On the other hand, the lowest crystallinities were observed for PVA10%/P3KBT2%E and PVA10%/P3KBT1%E, *ca*. 0.37 and 0.40, respectively. Excluding these points as uncertain, the crystallinities, as determined from the ratio of the area of the peak P2 and Δ*H*° value,^63^ vary between 0.45 and 0.57 (Table 1).

**Table 1:** Crystallinity as Determined from DSC Endothermic Peak P2 Using Δ*H*° = 138.6 J g^–1^^63^

cross-linking	PVA9% P3KBT0%	PVA9% P3KBT3%	PVA9% P3KBT4%	PVA9% P3KBT5%	PVA10% P3KBT0%	PVA10% P3KBT1%	PVA10% P3KBT2%	PVA10% P3KBT3%
H	0.55	0.52	0.52	0.50	0.57	0.56	0.55	0.52
E	0.46	0.45	0.47	0.51	0.47	0.40	0.37	0.57

The electrical conductivity of the nanofibrous samples containing P3KBT was investigated. The presence of this conductive polymer in the polymeric blends has been already shown to have a positive effect on the improvement of the electrical properties of the final construct.^65^

To measure the conductivity, a well-defined protocol was followed and samples were exposed to iodine vapors before the measurements.^66,67^ The reaction between iodine and the polymer chains is a form of doping, which introduces charged species into the material and increases its electrical conductivity. The longer the exposure time to iodine, the more polymer chains are doped, and as a result, the higher conductivity can be reached. The conductivity of the non-cross-linked samples was also checked.

**Figure 6:**
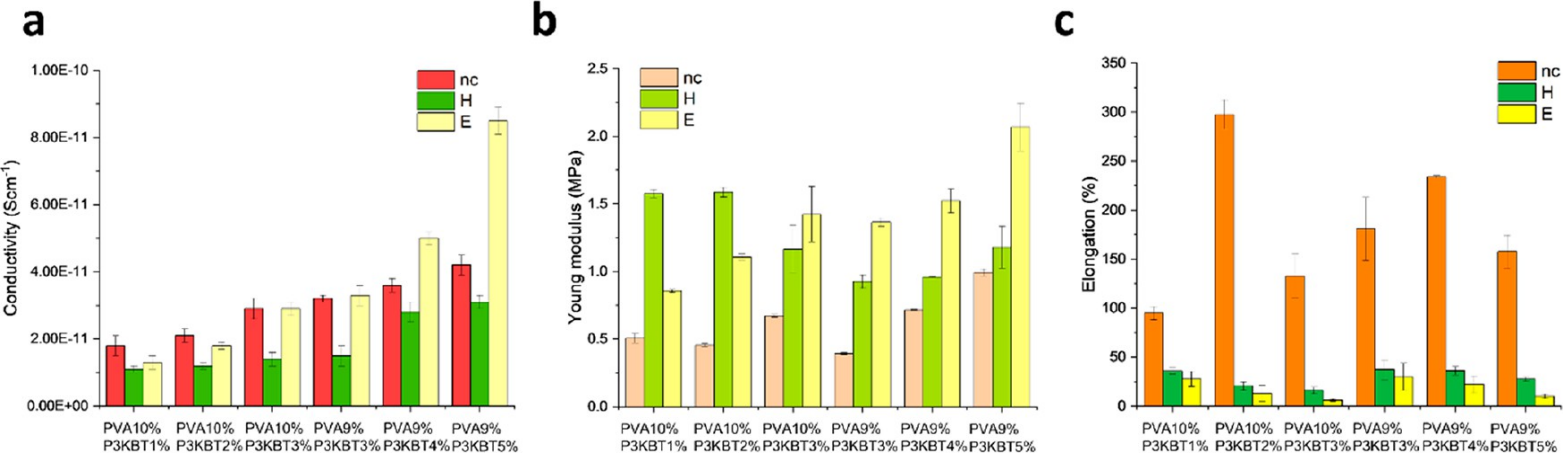
Electrical conductivity and mechanical properties of PVA/P3KBT systems. (a) Electrical conductivity of nc, H and E samples. (b) Young’s modulus for nc, H, and E samples. (c) Maximum percentage elongation of individual nanofibrous material samples before the breaking point.

Results showed that the cross-linking method had a significant effect on the conductivity of the samples (Figure 6a). Specifically, the samples cross-linked by method H had the lowest conductivity compared to both non-cross-linked and E-treated samples. This result may suggest that heat-induced cross-linking can lead to the formation of insulating networks that hinder charge transport.^68,69^

On the other hand, the E samples showed the highest conductivity compared to the other samples, except for the nc samples at lower P3KBT concentrations. This can be attributed to the fact that the ethanol treatment can promote the alignment of the PVA polymeric chains and leave behind a highly ordered structure, and subsequently, additional pathways for the conductive network of P3KBT would be accessible.^70,71^ Formation of intermolecular hydrogen bonding between the PVA and the P3KBT polymeric chains, an occurrence which is possibly eliminated during the thermal cross-linking, can be an extra positive benefit of the ethanol-based cross-linking method for attaining higher conductivity.

Furthermore, the conductivity of the E-treated samples increased with increasing P3KBT concentration, consistently with the well-known behavior of conductive polymers.^72^ Interestingly, it was found that the conductivity of the E-cross-linked samples increased with decreasing PVA concentration from 10% to 9%, which may be due to the reduced mechanical stability of the nanofibers and/or the formation of a more disordered and less conductive network.^73,74^ Moreover, a reduction in the number of insulating PVA chains can improve the charge transport in the semi-IPN. Finally, a decrease in the concentration of PVA can lead to the formation of thinner fibers with a higher surface area, which improves the contact between the fibers and therefore may play a role in the enhancement of the conductivity of the samples.^75^

In summary, the conductivity measurements demonstrated that the cross-linking of PVA/P3KBT systems via immersion in ethanol and subsequent thermal treatment resulted in enhanced conductivity which was directly proportional to the P3KBT concentration in the semi-IPN. However, the effect of PVA concentration on conductivity appears more complex and may depend on factors such as the P3KBT concentration and the specific cross-linking method employed.

Overall, obtained results suggest that the conductivity of nanofibrous PVA/P3KBT samples can be modulated by adjusting the P3KBT and PVA concentrations, as well as the cross-linking methods. These findings could have important implications for developing flexible and transparent conductive materials for a wide range of applications, such as organic electronics, sensors, and energy storage devices.

To assess the mechanical properties of the PVA/P3KBT systems, electrospun nanofibrous samples were subjected to tensile tests, based on which their stiffness and elasticity were determined. Both nc and cross-linked samples were tested to see if and how methods H and E affect the nanofiber properties.

The key parameter for determining the stiffness of materials is Young’s modulus, *i.e.*, the constant of linear elasticity, which determines the ratio of stress to the linear deformation of a solid. The calculated Young’s modulus values for each sample is plotted in Figure 6b. The first visible observation is the much lower value of the Young’s modulus for nc samples, indicating that they have definitively less stiffness than cross-linked samples. This can be explained by the fact that cross-linking, *i.e.*, arranging polymer chains in a regular network, creates more bonds between them, thus increasing stiffness. Moreover, stiffness increases for samples with a higher concentration of P3KBT (P3KBT/PVA > 2%), especially for the E-cross-linked samples. The PVA9%/P3KBT5%E nanofibers have the highest Young’s modulus, and therefore the highest stiffness, which corresponds perfectly with the lowest sorption capacity demonstrated in the swelling tests (as reported below). Nevertheless, all specific values of Young’s modulus are rather low and can be placed in the range of 0.4–2.1 MPa. This means that the tested materials are relatively soft and have a low resistance to deformation during external forces. One of the potential applications of electrospun nanofibers with obtained mechanical properties may be the production of biomedical materials like cardiovascular and neural implants or prostheses. In such applications, a material with mentioned Young’s modulus can help minimize tissue damage and increase user comfort. Young’s modulus indicates the material stiffness, *i.e.*, its resistance to deformation, but does not give information on how much stress the material can withstand before breaking.

In the second part of the research regarding the mechanical properties of the PVA/P3KBT nanoplatforms, the maximum length at break for each of the materials was analyzed. The elongation percentages of each sample are shown in Figure 6c. The results indicate that the non-cross-linked samples are the most elastic, and their stretching may result in elongation of about 100–300% compared to the initial length. The elongation at break of cross-linked samples is significantly lower and reaches nominal values, especially for E-treated samples; therefore, since these samples cannot be visibly elongated before breaking, their stress resistance is most probably very low. Nonetheless, it is worth noting that, from the point of view of specific applications, this type of material brings many benefits. Indeed, electrospun nanofibers with low elasticity often show high biocompatibility, *i.e.*, they are well tolerated by living organisms. Due to this, they can be used in medical applications, including implantology, or production of dressings. These nanofibrous systems can also be used for liquid or gas filtration because they have less tendency to stretch under the influence of the flowing medium. Additionally, they can also be used as protective materials against harmful radiation because they can form a dense mesh that will effectively block the penetration.

### Nanoplatform Behavior in Water

#### Water Solubility Tests

**Figure 7:**
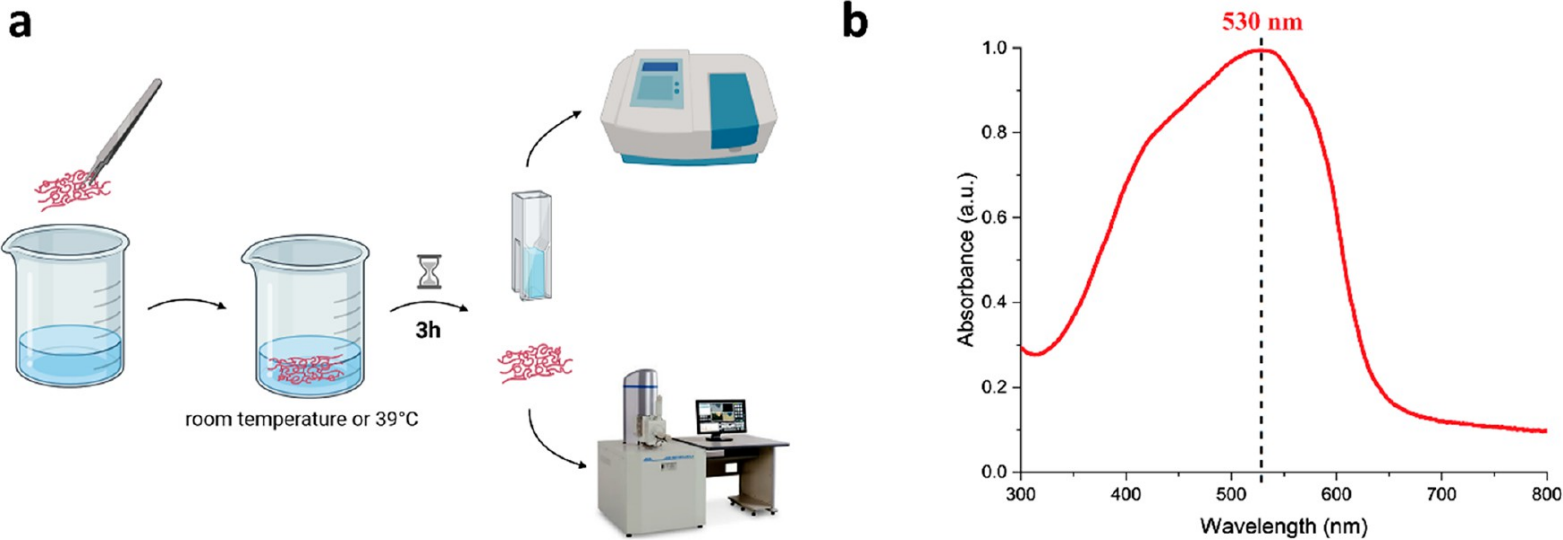
Tools for water stability testing and P3KBT release studies from semi-IPN systems. (a) Schematic presentation of the water solubility tests performed for nanofibrous samples. (b) Absorption spectrum of P3KBT in aqueous solution ([P3KBT] = 0.1 mg mL^–1^) with a peak maximum at 530 nm. The curve was normalized to 1.0. Each sample immersed in water at room temperature or 39 °C was air-dried after 3 h of testing and analyzed using SEM. The supernatants were subjected to spectrometric analysis to determine the amount of released P3KBT. The absorption maximum of P3KBT in an aqueous solution was determined as 530 nm.

In order to compare the effectiveness of the cross-linking methods, and to check the effect of the PVA and P3KBT polymer concentrations on the stability of the electrospun systems in water, solubility tests were carried out at room temperature and at 39 °C. The latter was chosen because its value is slightly higher than the body temperature of a healthy person, and testing the functionality of the developed systems in such conditions is crucial in terms of potential PVA/P3KBT nanoplatforms applications in the human body whose temperature can naturally fluctuate. The time of 3 h was sufficient to compare individual materials. When water was removed from vials with all the samples, they were allowed to dry in the air for subsequent SEM analysis, while the supernatants were retained for UV–Vis absorption measurements. A schematic representation of the operations carried out is shown in Figure 7a. SEM analysis allowed assessing the morphology of nanofibers after solubility tests, while spectroscopic analysis allowed the determination of the possible presence of released P3KBT in water. Because the absorption maximum for this polymer in an aqueous solution is 530 nm (as displayed in Figure 7b), the peak intensities in the expected radiation region were measured for each of the supernatants.

**Figure 8:**
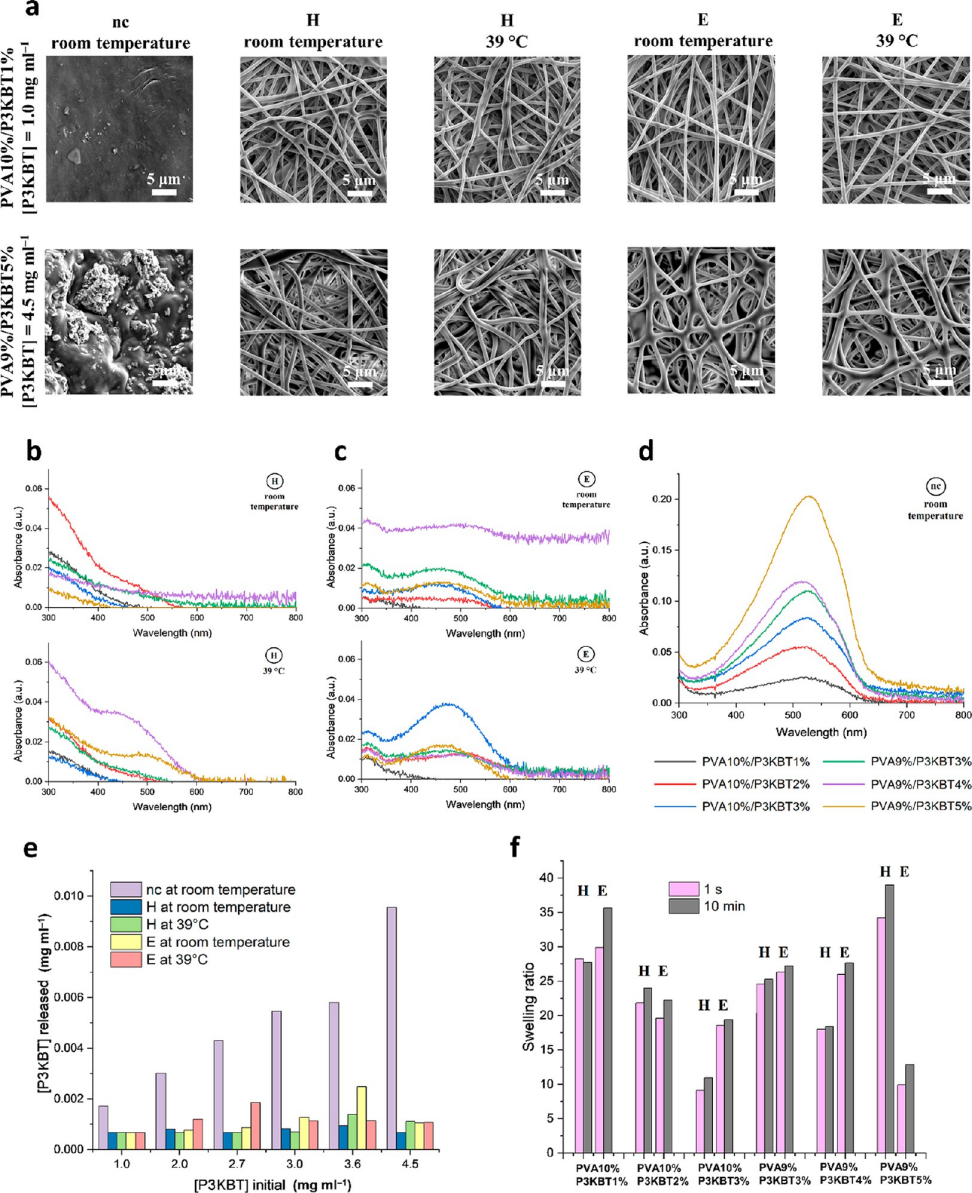
Water stability of PVA/P3KBT nanofibrous systems. (a) SEM images of nc, H, and E samples: PVA10%/P3KBT1% (containing the lowest tested concentrations of the polythiophene derivative in the fibers) and PVA9%/P3KBT5% (containing the highest tested concentrations of the polythiophene derivative in the fibers) after immersion in water at room temperature and at 39 °C for 3 h. (b) Absorption spectra of supernatants after solubility tests of H-cross-linked samples. (c) Absorption spectra of supernatants after solubility tests of E-cross-linked samples. (d) Absorption spectra of supernatants after solubility tests of nc samples. The legend applies to figure (b–d). (e) Comparison of the amount of P3KBT released into water from individual samples with different initial concentrations (*i.e.*, in precursor solution) as a result of their immersion in water at different temperatures for 3 h. P3KBT concentration of 1.0 mg mL^–1^ corresponds to the sample PVA10%/P3KBT1%, 2.0 mg mL^–1^ – PVA10%/P3KBT2%, 2.7 mg mL^–1^ – PVA9%/P3KBT3%, 3.0 mg mL^–1^ – PVA10%/P3KBT3%, 3.6 mg mL^–1^ – PVA9%/P3KBT4%, and 4.5 mg mL^–1^ – PVA9%/P3KBT5%. (f) Swelling ratio depending on the concentration of polymers in samples and the method of their cross-linking (H, E).

Figure 8a shows SEM images of samples with the lowest (PVA10%/P3KBT1%) and the highest (PVA9%/P3KBT5%) concentration of P3KBT among those tested, and also differing in the content of PVA. The SEM of the other conditions tested can be seen in Figure S4. In all cases, it can be clearly seen that the non-cross-linked nanofibers completely dissolved in water at room temperature. The surface of the analyzed samples is devoid of nanofibers; only certain residues or perhaps ordinary impurities are visible on them. In fact, the rapid dissolution of the nanofibers in water was visually observed at the very beginning of the experiment. PVA/P3KBTnc systems became invisible immediately after contact with water, and the water supernatant quickly turned into a pinkish color, typical for an aqueous solution of P3KBT. These results of the analysis are highly expected since both polymers present in the nanofibers are soluble in water. A different situation can be observed for cross-linked samples. Methods H and E allowed stabilization of the PVA/P3KBT systems by reducing their solubility in water. Regardless of the cross-linking method and the solubility test temperatures, all PVA10%/P3KBT1% samples retained their original form. SEM images do not show any morphological changes within the nanofibers. Samples with the highest concentration of P3KBT and a lower concentration of PVA (PVA9%/P3KBT5%) cross-linked by method H allow the same observations, supporting similar conclusions. Both at room and higher temperatures, the nanofibers did not dissolve, and their shape and thickness were preserved. However, in the case of method E, the obtained SEM images suggest that the morphology of the nanofibers has changed slightly, they are a bit fused and thicker, but it is still noteworthy that they did not dissolve (even at 39 °C) in contrast to the non-cross-linked systems. Furthermore, the increased thickness of the air-dried E hydrogel samples may point to an improved water retention capability. The conducted analysis allows drawing a preliminary conclusion that both cross-linking methods brought the desired stability.

#### Polythiophene Derivative Release Study

Spectrophotometric analysis has been proven to be helpful in confirming or disproving the preliminary conclusion regarding the H and E cross-linking efficiency. All supernatants were analyzed, searching for peaks (wavelength ≈530 nm) indicating the presence of P3KBT in the water.

Figure 8b shows the absorption spectra of the supernatants after the solubility tests of the H-treated samples. At room temperature, the P3KBT polymer was not released from the fibers into the aqueos medium, which is indicated by the lack of any peak in the analyzed spectral region. At the temperature of 39 °C, a small amount of P3KBT was released into the water only from two samples (containing the highest concentrations of P3KBT), *i.e.*, PVA9%/P3KBT4% and PVA9%/P3KBT5%. However, this amount is really minute. Observation of the absorption spectra on samples cross-linked by method E (Figure 8c) immediately reveals a greater number of scratched peaks than in the previous case (method H). At room temperature, a small amount of P3KBT was released into the water from four out of six samples. The nanofibers with the lowest concentration of polythiophene derivative, *i.e.*, PVA10%/P3KBT1% and PVA10%/P3KBT2%, remained intact. This situation changed at higher temperature, where only PVA10%/P3KBT1% did not present P3KBT leaking. However, again the amounts of P3KBT released were minimal. The confirmation of this statement can be found in Figure 8d, which shows the absorption spectra for supernatants after dissolution tests of nc samples. In this case, all samples have completely dissolved, so the peaks are sharp, and their intensity is much higher than in the cross-linked samples. Using the previously plotted calibration curve based on the amount of absorbance, the concentration of released P3KBT in each case was calculated and obtained results are shown in Figure 8e. After 3 h of testing, each non-cross-linked system released a large amount of P3KBT, significantly different from the amount released by the cross-linked samples. Samples H- and E-treated are highly stable in water; thus, they release negligible amounts of polythiophene derivative. Since our goal was to indicate the cross-linking method that allows obtaining the best results, as SEM analysis showed, it can be concluded that cross-linking by method H is more effective. Method E in each of the analyzed cases reported slightly less effective results; however, considering its properties, a more refined description of hydrogels can be established. Both E and H approaches showed highly satisfactory results, which allows for wide application of PVA-based fibrous hydrogels fabricated and cross-linked by the explained methods.

#### Swelling Ratio

The swelling tests of PVA/P3KBT nanofibers consisted in immersing the samples in water for a specific time, as a result of which the polymers absorbed the medium and increased in mass. The swelling ratio was defined and calculated as the ratio of the weight change of a sample due to swelling to its weight in a dry state. Tests have shown that when samples are immersed in water and then removed quickly (≈1 s), they absorb almost the maximum amount of water they are capable comparable to results obtained for later time points. In the following minutes, their mass slightly changes, but after 10 min, it reaches plateau, indicating that the absorption capacity has been achieved. The results presented in Figure 8f show that, depending on the material, the degree of swelling ranged from 10.94 (1094% for PVA10%/P3KBT3%H) to as high as 39.00 (3900% for PVA9%/P3KBT5%H). The nanofibers collected using the electrospinning technique, which were then subjected to stabilization processes, constitute nanofibrous hydrogels with a 3D structure similar to a network of bulk hydrogels. Compared to a bulk network, the undoubted advantage of nanostructuring of a hydrogel is its smaller diameter, large specific surface area, and better mechanical and hydrophilic properties. Increasing the pore size and permeability of the hydrogel allows for better diffusion of nutrients and waste products. Nanostructuring can also increase the hydrogel surface area, improving its ability to interact with other materials, such as cells or drugs. Additionally, the small size of the nanofibers used in the structure of the hydrogel can create a more densely packed network, which leads to more robust and stable hydrogel. The swelling tests confirmed high water capacity of these materials and their ability to absorb a significant amount of water. Nevertheless, comparing both cross-linking methods, it can be seen that within a given system, the degree of swelling often differs after using methods H and E. It can be explained by the fact that an increase in the degree of cross-linking of nanofibers increases the stiffness of the polymer network; therefore it is accompanied by a decrease in the degree of swelling.^76^

### Photothermal Responsivity under Solar Irradiation

**Figure 9:**
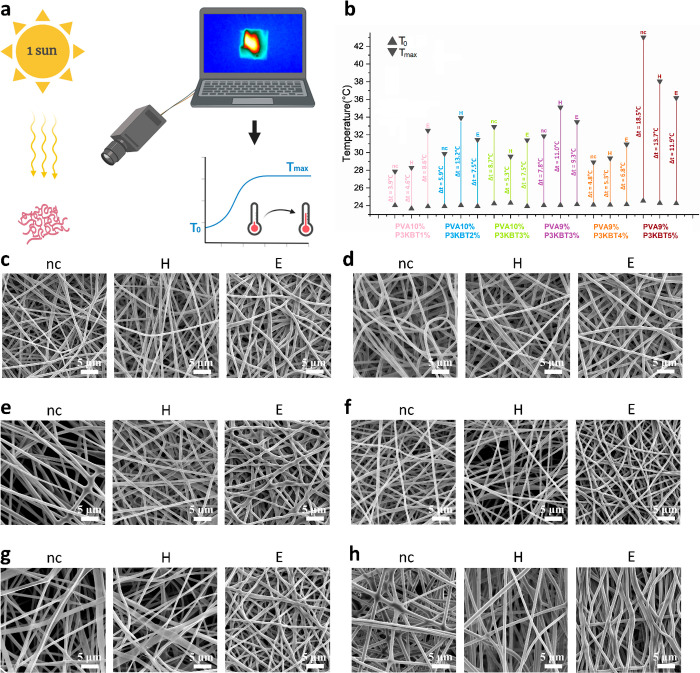
Photoresponsivity tests and SEM images of PVA/P3KBT nanofibers after exposure to artificial sunlight. (a) Sketch showing the experiment with the use of a solar simulator (radiation source – 1 kW m^–2^) and a thermal imaging camera, from which the image is displayed on a computer screen in real time. (b) Temperature changes of individual PVA/P3KBT systems in response to exposure to artificially generated solar radiation. (c) SEM images of PVA10%/P3KBT1% samples. (d) SEM images of PVA10%/P3KBT2% samples. e) SEM images of PVA10%/P3KBT3% samples. (f) SEM images of PVA9%/P3KBT3% samples. (g) SEM images of PVA9%/P3KBT4% samples. (h) SEM images of PVA9%/P3KBT5% samples. Temperature changes within all samples range from 3.9 to 18.5 °C. SEM analysis indicated that the increase in the system temperature did not cause any visible damage to the structure.

The presence of polythiophene-based polymer chains in the fibers (PVA/P3KBT semi-IPN) provided their photoresponsivity. The experiment was carried out using a solar simulator and a thermal imaging camera, which allowed registration of the temperature changes of all samples over time caused by exposure to artificial sunlight. The course of the present experiment is schematically shown in Figure 9a. The obtained graphs were used to calculate the temperature change (Δ*T*) in each case, *i.e.*, the difference between the maximum temperature after irradiation (*T*_max_) and the initial temperature of the nanofibrous sample (*T*_0_). Figure 9b shows a graph containing all the information collected. Temperature changes within all samples range from 3.9 °C (for the nc sample with the lowest polythiophene concentration) to 18.5 °C (for the nc sample with the highest polythiophene concentration). These results are definitely satisfactory, bearing in mind that the actual concentration of polythiophene in the fibers precursor solutions ranges from 0.10% to 0.45% (w/v). Indeed, in the case of non-cross-linked samples, a trend of greater temperature increase can be seen with increasing P3KBT concentration. However, there is no visible trend that would allow determining the dependence of temperature changes on the nanofibers cross-linking methods. After tests, all the samples exposed to light were analyzed using SEM to detect possible changes in the morphology of the nanofibers, which could potentially be disturbed or damaged by the raised temperature. SEM images shown in Figure 9c-h clearly indicate that the temperatures reached—in the best case even 42 °C—did not cause any visible damage to the nanofibers. This fact is extremely important from the point of view of considering a practical application for photoresponsive polymer systems, *e.g*., in stimuli-responsive wound dressings or photovoltaics.

### *In Vitro* Biological Studies

**Figure 10:**
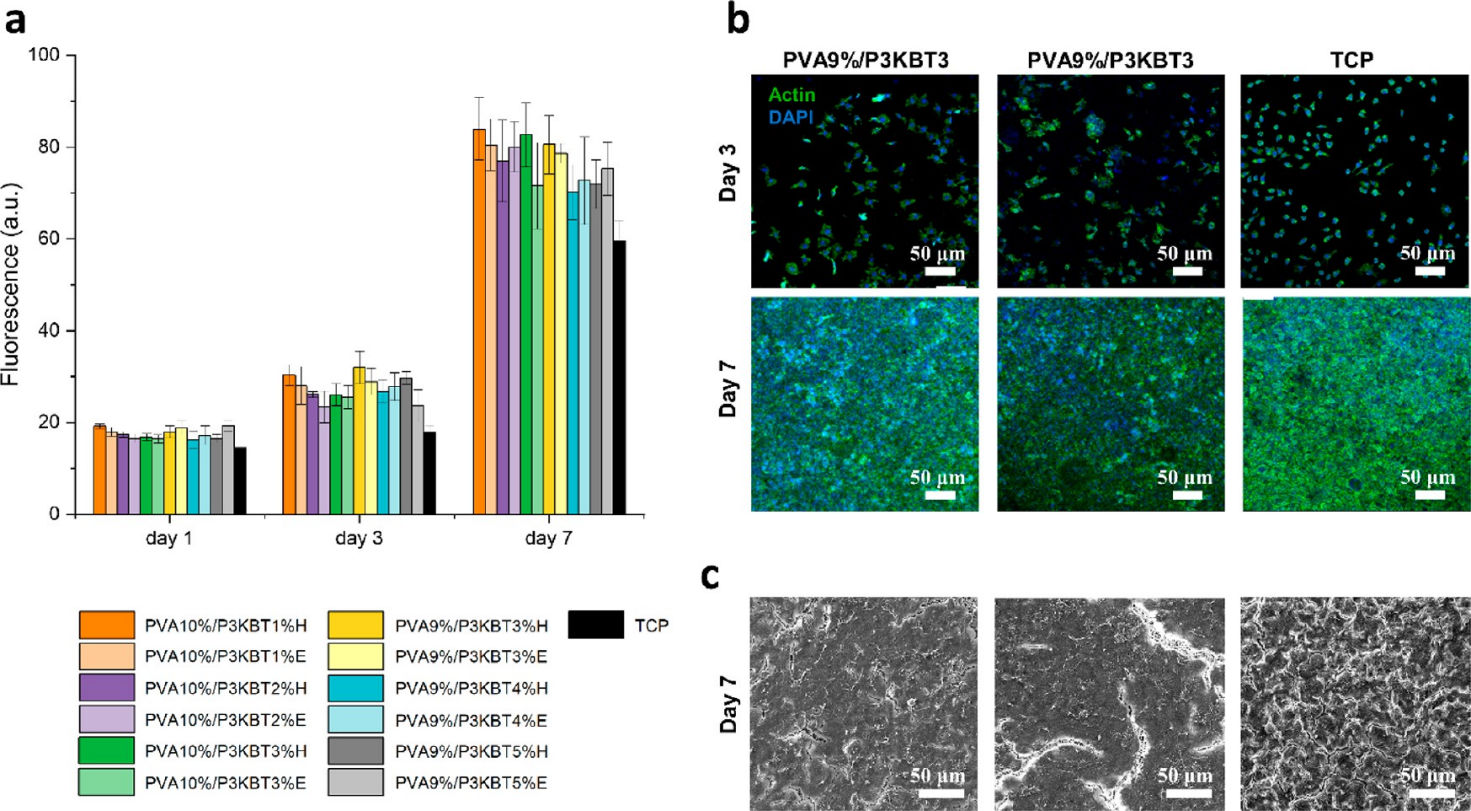
In vitro biological response: viability of L929 fibroblast cells and cell morphology of L929 fibroblasts seeded on PVA/P3KBT fibrous constructs cross-linked by ethanol immersion combined with heating or heating only and TCP. (a) Viability of L929 fibroblast cells seeded on PVA/P3KBT structures and TCP up to 7 days of culture. The increasing trend of viability during the culture time is visible in each tested condition. (b) Confocal images of samples stained with Actin (green color) for cell cytoskeleton visualization and DAPI (blue color) for individuating the cell nuclei. Scale bar: 100 μm. (c) SEM images of L929 fibroblasts cultured on fibrous constructs for 7 days compared to TCP control condition.

To evaluate the biological properties of the proposed fibrous matrices and verify their potential for biomedical applications, the material interaction with L929 fibroblast cells was investigated.^77,78^ The influence of different PVA and P3KBT concentrations was assessed, while possible effects of the cross-linking methods on cell response were analyzed. PVA concentrations of 9% and 10% were tested along with different concentrations of P3KBT. Samples were either cross-linked by method H or E. First, viability of L929 cells seeded on the samples was evaluated and compared to TCP control. A linear cell growth is reported in Figure 10a, showing increasing signals at each time point (1, 3, and 7 days) for all tested samples and control. No significant difference between the conditions was measured at any time points, confirming the biocompatibility of PVA and P3KBT, as demonstrated in previous studies.^65,79^ However, because PVA9%/P3KBT3% samples showed the most promising characteristics, cell morphological analysis was performed on this sample formulation after cross-linking by ethanol immersion combined with thermal treatment or heating only and compared to TCP control condition. Confocal images of Actin/DAPI stained samples revealed cell cytoskeleton and nuclei after 3 and 7 days of culture, highlighting elongated L929 fibroblasts with typical spindle shape at the initial stage, and subsequent cell proliferation and population of the whole matrix surface (Figure 10b). This is also supported by SEM images captured after 7 days from seeding (Figure 10c), where cell spreading and proliferation on the sample surface are well visible with no evident difference among the tested conditions. Results showed the biocompatibility of the fibrous structures, underlining their suitability for cell attachment, survival, spreading and proliferation.

## Conclusions

In summary, in this work, tools for optimal cross-linking of PVA nanofibers containing the addition of a polythiophene derivative (P3KBT) were presented. Conductive semi-IPN fibrous hydrogels were successfully produced by electrospinning precursor solutions with different concentrations of PVA and/or P3KBT and cross-linking by two “green” methods. The first method involved heating the nanofibers in an oven at 160 °C for 2 h, while the second method was based on stabilizing the nanofibers by immersing them in ethanol for 24 h followed by heating at 160 °C for 20 min. Both approaches are safe for the environment due to the lack of toxic or harmful chemicals. The article presents a detailed characterization of all the produced systems, which ultimately allows the selection of the optimal cross-linking conditions for their intended applications.

**Figure 11:**
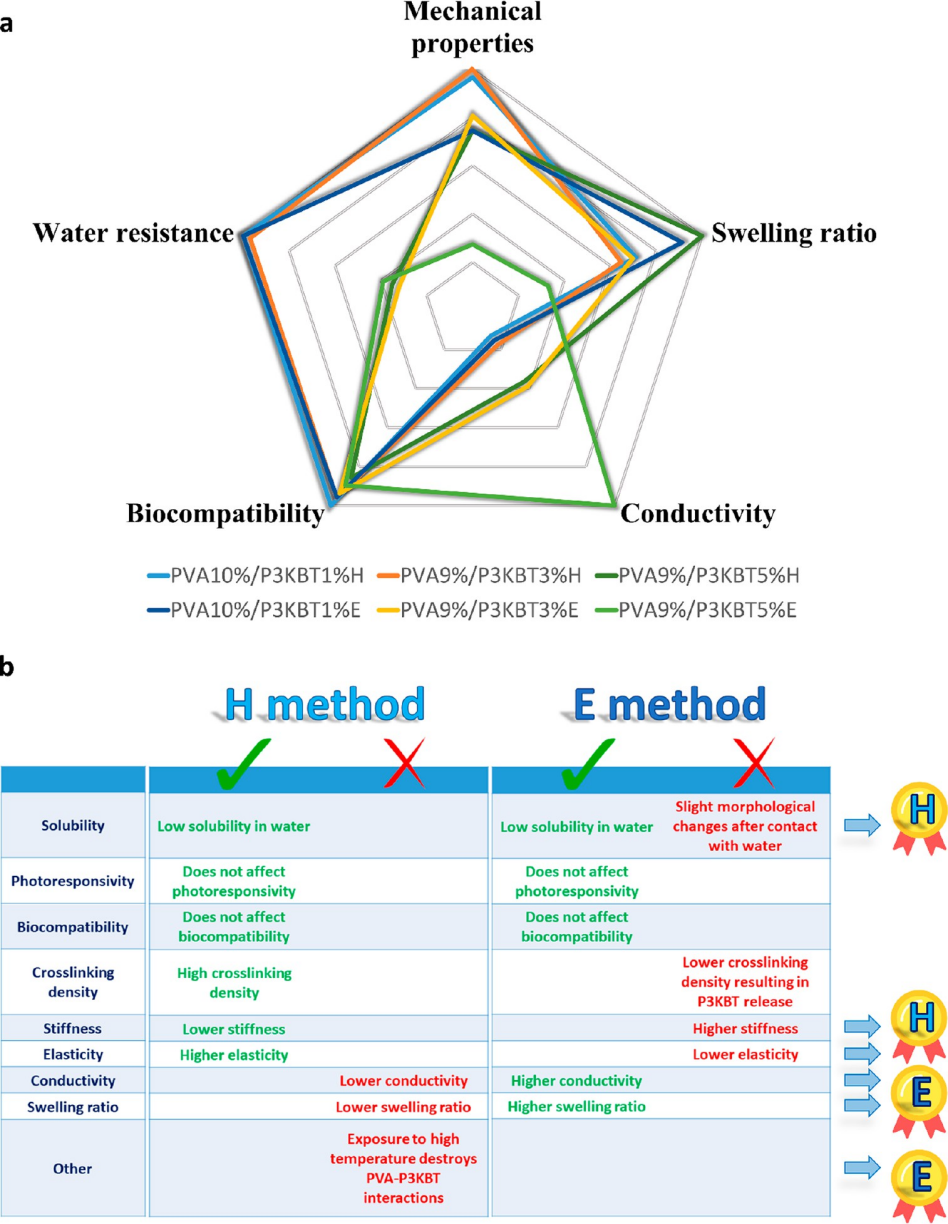
Summary of the results of all analyses performed. (a) Pentagonal diagram showing the characteristics of representative PVA/P3KBT systems depending on the polymer concentration and cross-linking method (H, E). Presented systems are nanofibers with the lowest and highest concentration of P3KBT among those tested, as well as intermediate nanofibers, characterized by potentially the best homogeneity. (b) Comparison of pros and cons of PVA/P3KBT systems cross-linked with two “green” approaches.

The morphological analysis confirmed that the nanofibers, both non-cross-linked and after cross-linking with methods H and E, retain a regular cylindrical shape and that their diameter is uniform within the sample. However, the increase in the diameter of cross-linked samples compared to as-spun systems indicates the formation of a more complex structure made of intermolecular connections, especially under the influence of E treatment. Further changes within the E-cross-linked samples were observed after contact with water. Water solubility tests have shown that both tested methods reduce the solubility of nanofibers, although method H is slightly more effective. In the case of method E, a greater amount of P3KBT is released from the semi-IPN in water, which is also accompanied by an increase in the diameter of the nanofibers. Nevertheless, in the method H, the samples were exposed to high temperature for longer than in the method E, which destroyed the PVA-P3KBT interactions and caused the redshift of the adsorption spectra of solid systems. In both H and E cases, the FT-IR results confirmed the formation of specific bonds within a given system, indicating successful PVA cross-linking. Moreover, spectra showed an increase in the degree of crystallinity of the cross-linked samples, and a greater reduction in the −O–H stretching peak after using method H, evidence its higher efficiency compared to method E. The DSC results also confirm these observations. H-cross-linked samples are characterized by greater crystallinity, and a decrease in the melting point indicates a higher cross-linking density. Interestingly, during the tests, the photoresponsivity of the systems was demonstrated, which turned out to be not affected by the cross-linking method but by the concentration of P3KBT that reacts to sunlight. In contrast, the conductivity of the analyzed systems depends on the cross-linking method. The electrical conductivity is, as expected, greater the higher the concentration of conductive P3KBT, but for the same samples subjected to the E treatment, it reaches higher values than those for the method H. This fact once again indicates a higher degree of cross-linking of thermally treated nanofibers, whose dense network acts as an insulator that prevents the free flow of electric charges. Importantly, the proven photoresponsive and conductive properties pave the way for further research and encourage attempts to improve them using previously explored approaches, such as the incorporation of plasmonic materials, *e.g*., gold and silver nanoparticles, respectively. Studies on the interaction of semi-IPNs with water have shown that nanofibrous hydrogels are characterized by a high water and moisture absorption capacity. In addition, in the first seconds after water immersion, they absorb practically the maximum amount of water they can intake. In as many as four out of six cases, the samples cross-linked by the method H swell slightly less than the samples cross-linked by the method E. This can be explained by the stiffer polymer structure of the thermally treated samples, and thus the increase in cross-linking compared to the E systems. Moreover, the higher degree of E sample swelling corresponds to their lower degree of crystallinity. Studies on the mechanical properties have shown that the E-treated samples increase their stiffness the higher the concentration of P3KBT contained in them. Additionally, this stiffness is higher than that for H samples, so they are characterized by lower flexibility. It is therefore considered that the samples cross-linked using the method H have better mechanical properties and are more resistant to external forces. All analyzed systems are biocompatible. The entire characterization carried out indicates the effectiveness of both methods of PVA cross-linking. H and E approaches can be successfully used and adapted to the potential application of PVA-based nanofibrous systems. Collected data are summarized in Figure 11.

## Supporting Information Available

SEM images of pure electrospun PVA nanofibers differing in concentration, degree of hydrolysis, and pH; electrospinning conditions for pristine PVA and PVA/P3KBT solutions; content of PVA and P3KBT polymers in the precursor solutions and the P3KBT/PVA ratio in the analyzed samples; SEM images of PVA 10%, DH 99+% samples immediately after cross-linking under seven different conditions, as well as after solubility tests in water at room temperature (24 h) and 39 °C (3 h); FT-IR spectrum of pure 9% PVA nanofibrous material before and after cross-linking by methods H and E; SEM images of nc, H, and E samples after immersion in water at room temperature and at 39 °C for 3 h; method of normalizing the results shown in Figure 11a (PDF)
