# Supplementary material for: Electrospun Poly(vinyl alcohol)-Based Conductive Semi-interpenetrating Polymer Network Fibrous Hydrogel: A Toolbox for Optimal Cross-Linking
Source: ACS Mater Au. 2023 Jun 8;3(5):464–82. doi: 10.1021/acsmaterialsau.3c00025 (PMC10510526; doi:10.1021/acsmaterialsau.3c00025)
Supplement: Supplementary file 1 — mg3c00025_si_001.pdf [file mg3c00025_si_001.pdf]

Supporting Information

Electrospun poly(vinyl alcohol)-based conductive semi-interpenetrating polymer network fibrous hydrogel: a toolbox for optimal crosslinking

Anna Zakrzewska,[†] *Seyed Shahrooz Zargarian,*[†] *Chiara Rinoldi,*[†] *Arkadiusz Gradys,*[§] *Dariusz Jarząbek,*[⊥] *Michele Zanoni,*[‖] *Chiara Gualandi,*[‖] *Massimiliano Lanzi,*[‡] *and Filippo Pierini*[†]*

[†]Department of Biosystems and Soft Matter, Institute of Fundamental Technological Research, Polish Academy of Sciences, Pawińskiego 5B, 02-106 Warsaw, Poland

[§]Laboratory of Polymers and Biomaterials, Institute of Fundamental Technological Research, Polish Academy of Sciences, Pawińskiego 5B, 02-106 Warsaw, Poland

[⊥]Department of Mechanics of Materials, Institute of Fundamental Technological Research, Polish Academy of Sciences, Pawińskiego 5B, 02-106 Warsaw, Poland

[‖]Department of Chemistry "Giacomo Ciamician", University of Bologna, Via Selmi 2, 40126 Bologna, Italy

[‡]Department of Industrial Chemistry "Toso Montanari", University of Bologna, Viale del Risorgimento 4, 40136 Bologna, Italy

*Corresponding author e-mail address: fpierini@ippt.pan.pl

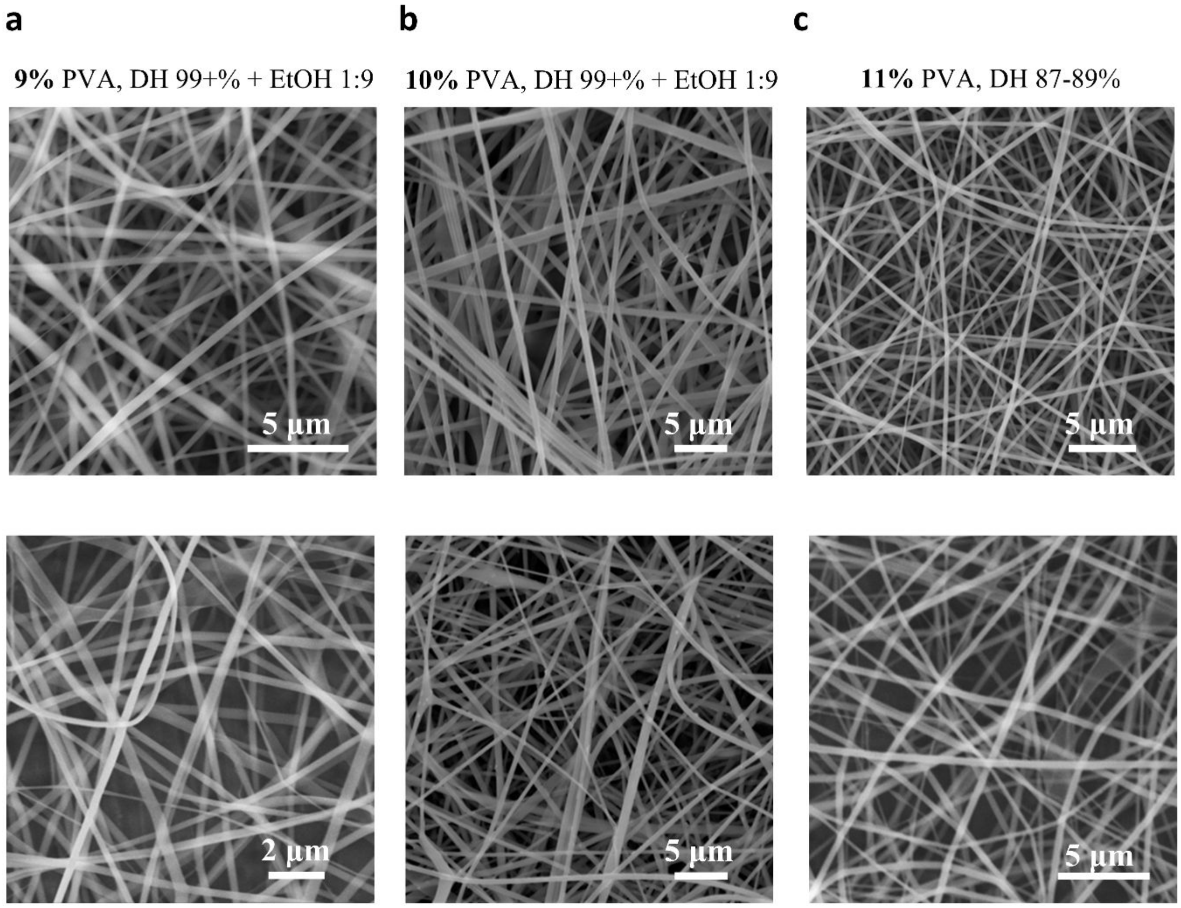

Figure S1. SEM images of pure electrospun PVA nanofibers differing in concentration, degree of hydrolysis, and pH. a) PVA 9%, DH 99+%, pH 7 and pH 3. b) PVA 10%, DH 99+%, pH 7 and pH 3. c) PVA 11%, DH 87 – 89%, pH 7 and pH 3. pH 3 was obtained by acidifying the precursor solution with hydrochloric acid (HCl). This was intended to provide an acid catalyst for the glutaraldehyde vapor crosslinking process, which was later tested. Due to the morphology and properties of nanofibers dependent on DH, mats obtained in accordance with conditions a) and b) pH 7 were selected for further research.

Table S1. Electrospinning conditions for pristine PVA and PVA/P3KBT solutions: distance between the tip of the needle and collector surface, applied voltage, and solution flow rate.

PRECURSOR SOLUTION	DISTANCE	VOLTAGE	FLOW RATE
**PVA 10%/P3KBT 0.00%**	12 cm	15 kV	500 µl/h
**PVA 10%/P3KBT 0.10%**	14 cm	15 kV	500 µl/h
**PVA 10%/P3KBT 0.20%**	14 cm	15 kV	500 µl/h
**PVA 10%/P3KBT 0.30%**	14 cm	15 kV	500 µl/h
**PVA 9%/P3KBT 0.00%**	12 cm	15 kV	500 µl/h
**PVA 9%/P3KBT 0.27%**	15 cm	14 kV	500 µl/h
**PVA 9%/P3KBT 0.36%**	15 cm	13 kV	500 µl/h
**PVA9%/P3KBT 0.45%**	15 cm	13 kV	500 µl/h

Table S2. The content of PVA and P3KBT polymers in the precursor solutions and the P3KBT/PVA ratio in the analyzed samples.

SAMPLE CODE	$[PVA]_{SOLUTION}$ (W/V)	$[P3KBT]_{SOLUTION}$ (W/V)	$P3KBT/PVA_{SOLUTION}$ $P3KBT/PVA_{NANOFIBERS}$
**PVA10%/P3KBT0%**	10%	0.00%	0/99 → 0%
**PVA10%/P3KBT1%**	10%	0.10%	1/99 → 1%
**PVA10%/P3KBT2%**	10%	0.20%	2/98 → 2%
**PVA10%/P3KBT3%**	10%	0.30%	3/97 → 3%
**PVA9%/P3KBT0%**	9%	0.00%	0/99 → 0%
**PVA9%/P3KBT3%**	9%	0.27%	3/97 → 3%
**PVA9%/P3KBT4%**	9%	0.36%	4/96 → 4%
**PVA9%/P3KBT5%**	9%	0.45%	5/95 → 5%

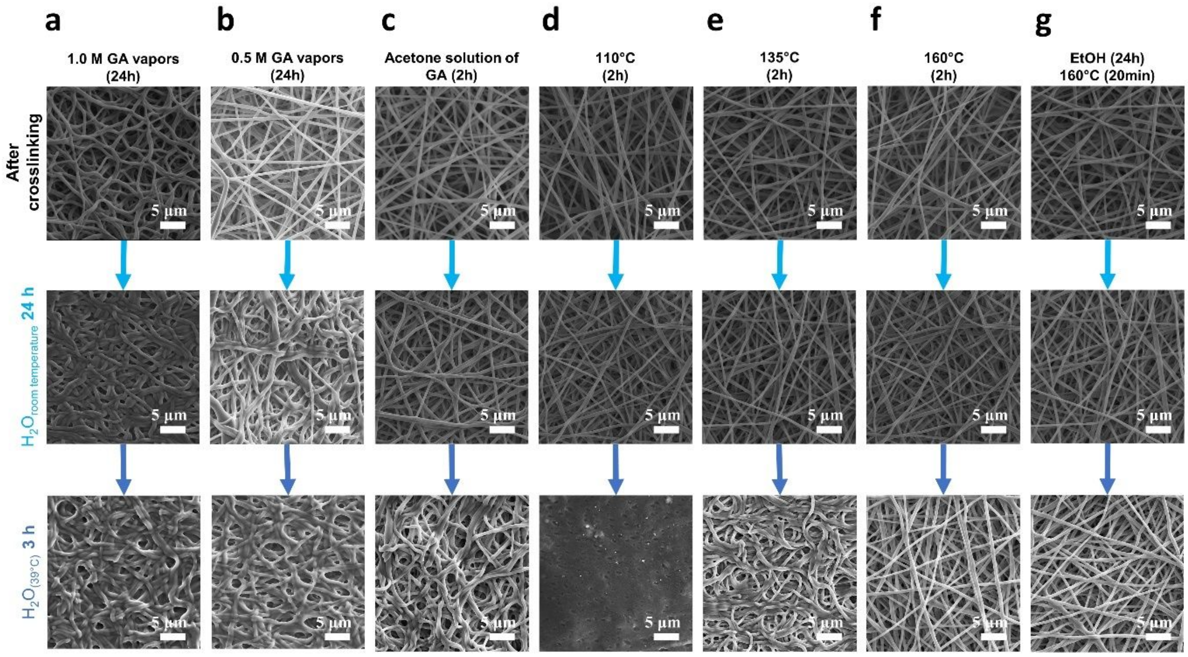

Figure S2. SEM images of PVA 10%, DH 99+% samples immediately after crosslinking under seven different conditions, as well as after solubility tests in water at room temperature (24h) and 39 °C (3h). In both analyzed cases, the original morphology of the nanofibers was preserved only for the fibers crosslinked under conditions shown in Figures f) and g). The methods of temperature crosslinking (160 °C, 2h) and crosslinking by immersion in ethanol (24h) followed by thermal treatment (160 °C, 20 min) were found to be the most effective and selected for further analysis.

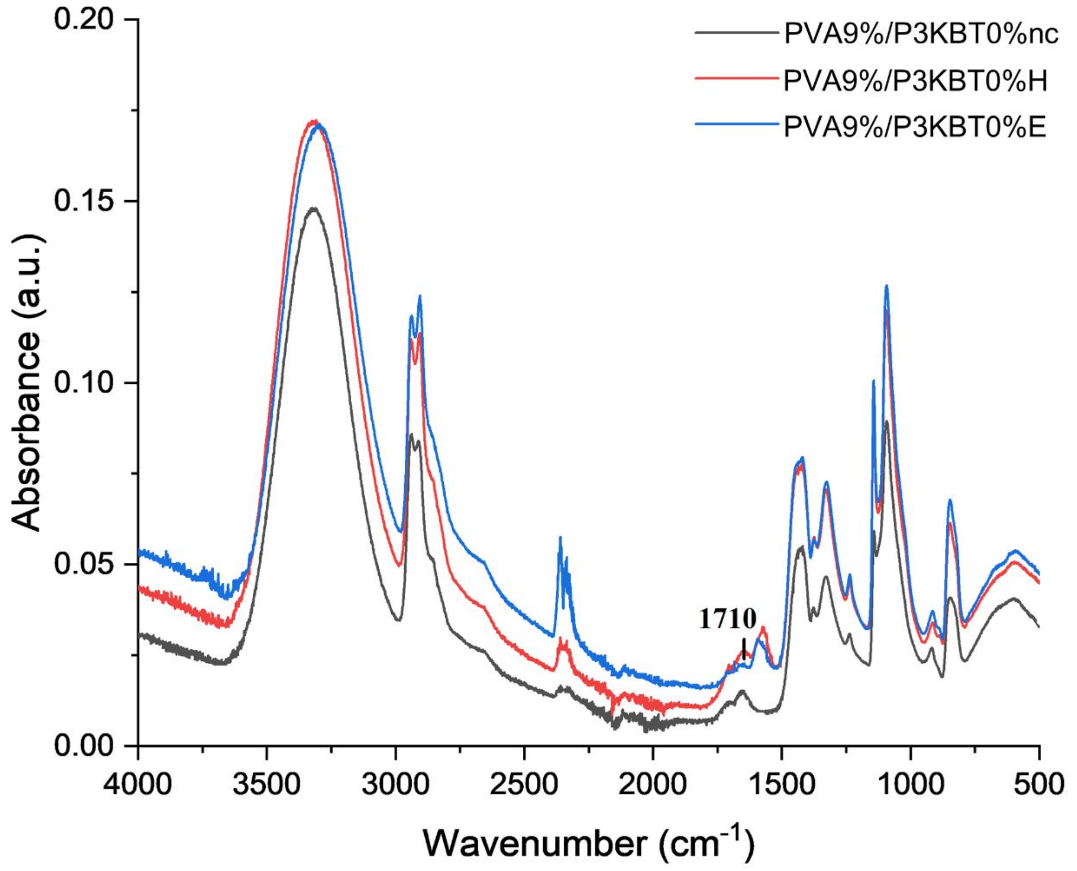

Figure S3. FT-IR spectrum of pure 9% PVA nanofibrous material (PVA9%/P3KBT0%) before and after crosslinking by methods H and E.

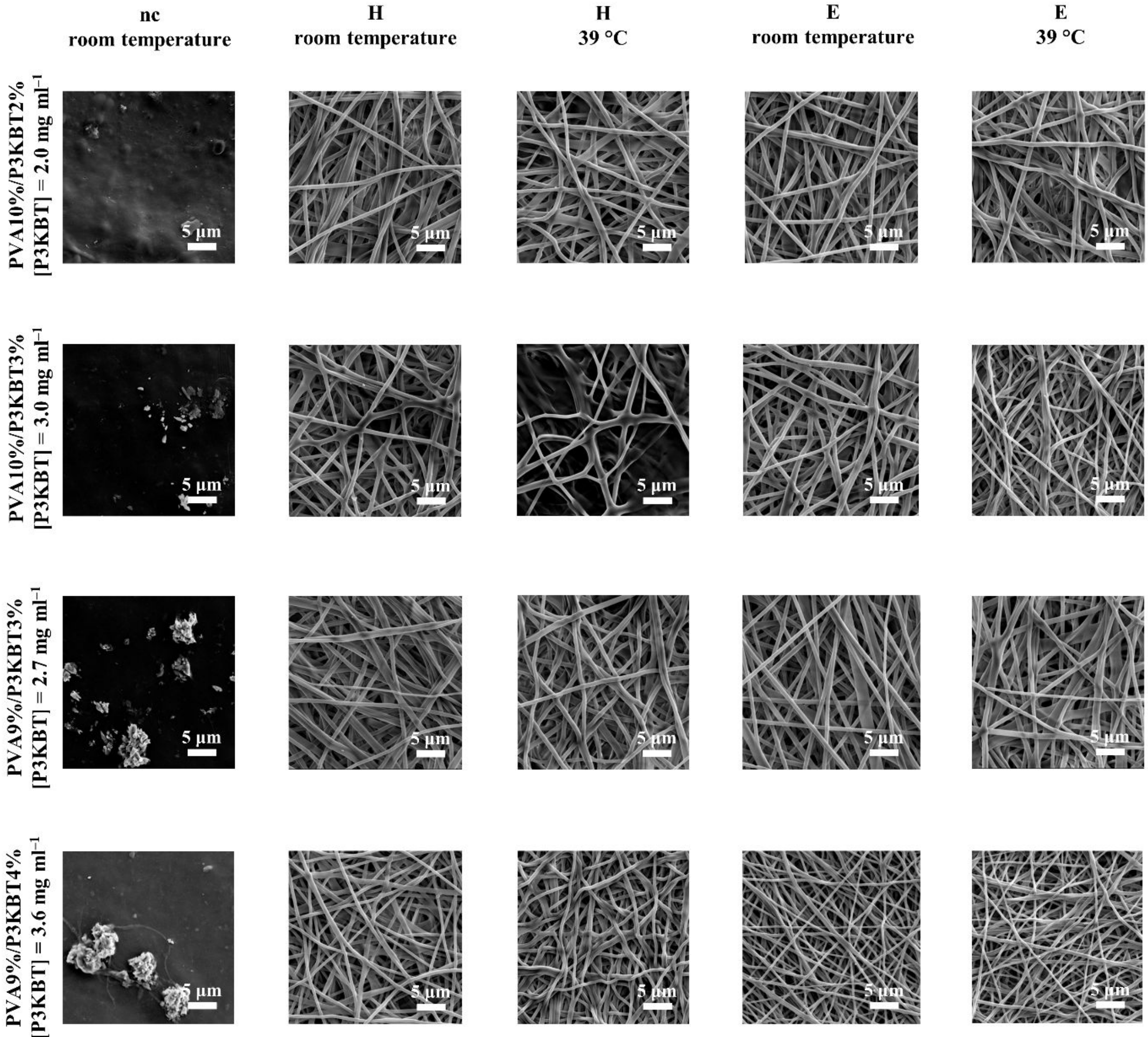

Figure S4. SEM images of nc, H, and E samples after immersion in water at room temperature and at 39 °C for 3h.

Method of normalizing the results shown in Figure 11a.

Figure 11 is a summary of the main results obtained during the performed analyses, allowing the comparison of both tested crosslinking methods (H, E). The pentagon diagram shown in Figure 11a was prepared by collecting data representing a given property, i.e.:

- Percentage elongation to break for "Mechanical properties";
- Swelling degree of samples placed in water for 10 min for "Swelling ratio";
- Electrical conductivity for "Conductivity";

- Viability of L929 fibroblast cells after 7 days for "Biocompatibility";
- Amount of P3KBT released into the water at 39 °C in 3h for "Water resistance".

The values representing each property were normalized so that the most desirable value became 1.0, and the others are correspondingly lower.
